# A Qualitative Analysis of How Underage Adolescents Access Nicotine Vaping Products in Aotearoa New Zealand

**DOI:** 10.1093/ntr/ntae096

**Published:** 2024-04-20

**Authors:** Katie Frost, Anna Graham-DeMello, Jude Ball, Michaela Pettie (Ngāti Pūkenga), Janet Hoek

**Affiliations:** Otago School of Medicine, University of Otago, Dunedin, New Zealand; Department of Preventive and Social Medicine, University of Otago, Dunedin, New Zealand; Department of Public Health, University of Otago, Wellington, New Zealand; Department of Public Health, University of Otago, Wellington, New Zealand; Department of Public Health, University of Otago, Wellington, New Zealand

## Abstract

**Introduction:**

Despite policies setting a minimum legal sales age, youth continue to access electronic cigarettes (ECs). Evidence of rising youth vaping prevalence in many countries suggests existing measures have serious loopholes and raise important questions about how youth source vaping products.

**Aims and Methods:**

We explored how youth source ECs using in-depth interviews with 30 adolescents aged 16–17 who vaped at least once a month and lived in Aotearoa New Zealand. Our semistructured interview guide probed participants’ vaping experiences and how they developed and used social, quasi-commercial, and commercial supply routes to access ECs. We used an inductive reflexive thematic analysis approach to interpret the data.

**Results:**

Nearly all participants shared ECs with peers and sharing was the sole access route for some. Many used proxies, often older relatives or people they knew socially, to purchase ECs on their behalf; however, others recruited proxies by approaching previously unknown people they identified on social media. Participants also sourced ECs via quasi-commercial networks that existed within schools and on social media, and some purchased in their own right, usually from smaller retail outlets that did not ask for ID.

**Conclusions:**

Disrupting social supply will be challenging, though reducing ECs’ availability, appeal, and affordability could make social supply, including sharing and proxy purchasing, more difficult. Reports that youth purchase ECs from commercial retailers known to waive age verification suggest stronger monitoring and enforcement, along with escalating retailer penalties, are required.

**Implications:**

Vaping access routes sit on a continuum from informal, spontaneous sharing to carefully planned commercial purchases. While supply via friends, siblings and other social contacts is an important means of access, nicotine dependence drives some to use riskier access routes, including approaching unknown people to act as proxy purchasers. Evidence young people identify noncompliant retailers suggests policy makers should monitor and enforce existing measures more stringently and consider additional penalties for recidivist underage suppliers. A more comprehensive response that reduces the appeal, addictiveness, affordability, and availability of vaping products would address factors fostering and maintaining youth vaping.

## Introduction

Internationally, e-cigarette (EC) use, or “vaping,” among adolescents has increased rapidly, particularly since refillable pod mods (eg, JUUL) and disposable devices (eg, Elf bars) became available. These products have high visual appeal for youth (due to their sleek designs and attractive, colorful packaging), arouse curiosity, and deliver high concentrations of nicotine (a highly addictive chemical) in a very palatable format.^1–3^

Possible health risks for youth EC users include immune system suppression,^4^ acute cardiovascular stress,^5^ respiratory issues,^6^ and worsening of asthma symptoms.^4,7^ Those who use nicotine-containing ECs also risk becoming addicted, and may experience psychological stress and reduced well-being as a result.^8^ In addition, some studies have found there to be a greater likelihood of subsequent cigarette smoking among youth EC users as compare to nonusers^9–11^; others have found an inverse relationship between youth vaping and smoking and dispute this association.^9^

Policy makers in many high-income (or “developed”) countries have tried to prevent EC uptake among young people by setting a minimum legal sales age of 18^12^ or 21,^13^ mandating on-pack warnings, and restricting flavors that appeal to young people.^14^ Australia has gone further by classifying nicotine-containing ECs not as consumer products but as therapeutic products available only by prescription.^15^

Yet despite these efforts, youth EC use has continued to increase in Canada,^16^ the United Kingdom,^17,18^ Australia,^19,20^ and in Aotearoa New Zealand (henceforth Aotearoa NZ), where the 2021/2022 NZ Health Survey (NZHS) found 14% of 15- to 17-year-olds had vaped in the past month (compared to 0.6% in 2015/2016).^21^ However, these data do not capture profound disparities in use prevalence (and therefore risk burden) among young people within these countries. In Aotearoa NZ, for instance, the 2022 Snapshot Year 10 Survey found that Māori youth aged 14–15 engage in “regular” (ie, past month) vaping at more than twice the rate of their European/Pakeha (White New Zealander) counterparts (33.8% vs. 15.7%, respectively).^22^

Although the US National Youth Tobacco Survey (NYTS) reported declining EC use prevalence in 2020, it found further increases between 2021 and 2022,^13^ and overall prevalence remained high. The Centers for Disease Control and Prevention (CDC) has warned against comparing post-2020 NYTS findings with previous survey waves, as COVID-19 protocols led to significant changes in data collection methods. Interpretation of recent results thus requires caution and ongoing monitoring is needed, with a trend in use prevalence yet to be confirmed.^13^

Nonetheless, evidence that underage youth continue to access ECs, despite the measures put in place, raises important questions about the supply channels they use and how these operate. US, Canadian, English, and Australian studies indicate underage youth typically access ECs using social sources,^23–30^ with schools and social events offering ample opportunity for experimentation and ongoing use.^31,32^ Commercial self-purchasing from brick-and-mortar retailers also occurs among underage youth,^26,33,34^ raising questions about retailer compliance with age verification policies. However, while studies have identified some access routes young people use, we know little about how these evolve and operate, or how future policies could close regulatory gaps. We explored these knowledge gaps by probing how young people developed and used social, quasi-commercial, and commercial supply routes to access ECs.

## Methods

### Participants

We report on two separate samples of eligible young people aged 16 or 17 who vaped at least once a month, lived in Aotearoa NZ, and could participate in an in-person or online interview exploring EC access.

#### Sample 1

JH and KF recruited 13 participants using social and community media, personal networks, snowball sampling in Dunedin, a provincial city in Aotearoa NZ. Because nonvalid responses disrupted recruitment, AG-DM enrolled a further nine participants from University of Otago residential colleges. Interested people could obtain information via phone or e-mail, or proceed to an online eligibility survey where they provided demographic information and responded to questions about their vaping habits (Supplementary File 1). All participants gave verbal consent and received a $30 voucher as a token of thanks.

#### Sample 2

JB and MP recruited 64 high-school students from Wellington as part of the Adolescent Friendships and Lifestyles 2.0 (AFL 2.0) project, which investigated substance use; eight of these students met the current study eligibility criteria. JB and MP selected students randomly from the school roll and invited them to participate; recruitment was also undertaken using snowball sampling and teacher referrals. All participants and their caregivers gave written informed consent and participants received a $20 voucher as a token of thanks.

The total sample comprised 30 participants. The University of Otago Human Ethics Committee reviewed and approved each project (references D22/300 and 22/017), and the combined data approach used. Supplementary File 2 presents participant flowcharts for each sample and additional recruitment information, including promotional materials used on social media, details about nonvalid responses (Sample 1), and school-level recruitment priorities and processes (Sample 2).

### Data Collection Procedures

#### Sample 1

KF, JH, and AG-DM conducted individual interviews online (using Zoom) between November 2022 and March 2023. We probed vaping experiences and product access using a semistructured interview guide; underage participants living in university residential colleges were asked to reflect on their high-school experiences, just a couple of months prior, when responding (see Supplementary File 2 for further details).

#### Sample 2

JB, MP, and a student interviewer, Loleseti Poasa, conducted interviews in-person, between June and October 2022. The semistructured interviews explored perceptions of and experiences with ECs. Participants were initially interviewed in friendship pairs, which allowed peer group norms to surface, then in individual follow-up interviews, where they could share experiences they may not have revealed in the presence of a friend.

Across both samples, interviews lasted between 30 and 112 minutes. With participants’ permission, we audio-recorded all interviews and transcribed these into verbatim records; all participants were assigned a pseudonym. For Sample 1, participants received a copy of their transcript and summary notes and could comment and edit these (none did). Supplementary File 3 presents interview guides, including questions and probes used.

### Data Analysis

We interpreted the data using an inductive reflexive thematic analysis approach^35^ that recognized each researchers’ expertise and perspective.^36^ We wrote summary notes and analytical memos following interviews to interpret participants’ reflections, reflect on our own positions, and identify ideas for probing in subsequent interviews.

KF and JH developed a preliminary latent coding framework based on three transcripts,^36^ before meeting with AG-DM to review and develop its components. KF used NVivo 20 (Release 1.7.1) to code 10 more transcripts using this framework and met regularly with JH to discuss and review the work. JH and AG-DM reviewed all coding, undertook minor nuancing to accommodate additional Sample 2 transcripts, and developed an updated framework that AG-DM then used NVivo 20 (Release 1.7.1) to code 23 transcripts (the nine she undertook and a total of 14 friendship [*n* = 6] and individual [*n* = 8] interviews from the AFL 2.0 study). All authors met regularly to discuss findings and potential themes.

## Results

We first outline participants’ characteristics (Table 1) and summarize how their vaping practices evolved, before presenting three access-specific themes: Sharing (“passing around” vapes while spending time with peers, implying opportunistic access); Social Purchasing (enlisting others, often older peers, as proxy purchasers); and Commercial Self-Purchasing (via retailers). To reflect participants’ language, we refer to “vaping,” rather than “EC use,” but consider these terms interchangeable. Supplementary File 4 presents a detailed codebook.

**Table 1.** Participant Characteristics

Characteristic	Number
Age	
16	11
17	19
Gender	
Girls/women (F)	23
Boys/men (M)	7
Gender diverse	0
Ethnicity	
New Zealand European (NZE) or Other European (OE)	20
Māori	5
Pacific	3
Asian	1
Indian	1

### Evolution of Vaping Practices

Nearly all participants first experimented with vaping socially, where vaping’s visibility and pervasiveness made it appear normal and trial occurred organically. Anton (M, 16, OE) explained: “We were in the hallway at school… one of my mates gets out their vape… I thought, ‘Why not try it’?.... Everyone at school seems to do it.” Like Anton, many vaped to satisfy curiosity, affiliate with social groups, and reduce the fear they would miss out. Zoe (F, 16, Māori) commented: “[It] was a great social thing to have around me that I could just vape and talk to anyone… a bonding thing for me and my friends.” While most participants resisted the idea that peer pressure had prompted them to vape, some felt that by turning down opportunities to share a friend’s vape they could seem juvenile and disconnected from others. Lizzy (F, 17, NZE) felt: “…like it was almost… not embarrassing if you didn’t, but, you know, I wanted to not seem… childish.”

Participants enjoyed physical sensations from vaping: “headies” created “euphoria” and “dizziness” similar to inebriation, and some enjoyed performing tricks with vape aerosol. These appealing sensations, the ubiquity of vaping, and beliefs vaping helped participants manage stress and anxiety, encouraged continued use. Some found nicotine dependence occurred quickly and left them with little control over their vaping. Keira (F, 17, NZE/Māori) explained: “I went from not needing it to needing it within a very short time… like a month or two. Once I started feigning *[having strong cravings]*, I knew it was bad... If I’m anxious, I’ll have one and it fixes it… *[The fix is]* immediate, but temporary... I wish I could quit, but it’s just everywhere...” However, a minority controlled their vaping by actively monitoring and reining in their use, cognizant of people they knew who had become dependent. Selma (F, 17, NZE) explained: “It’s every time I’m drinking… just on the Thursday, Friday, Saturday. I’ve been conscious about not doing it outside of those times. Seeing my brother get addicted… it made me realize how serious it is. If this is happening to 13-year-olds, I need to make a conscious effort not to.”

Overall, participants saw vaping as highly normative, which had predisposed their own experimentation, and left several feeling heavily dependent on nicotine. We next probe supply routes participants used and how these had embedded vaping in their daily lives.

### Sharing: A Rampant and Opportunistic Social Phenomenon

All participants reported sharing vapes, typically with close friends, but also with family members, work acquaintances, or “randoms” who they encountered socially. Although sharing commonly took place at school, it was rampant at parties and other social gatherings, where it connected participants to social groups. Keira (F, 17, NZE/Māori) commented: “We all try each other’s and share. I can’t even explain how big it is… that’s the majority of it… If I’m with my friends, I’m probably not using my vape, I’m using theirs...”

Many participants shared other people’s vapes, which avoided the need to own a device and reinforced their identity as “social” or intermittent vapers who could control their use. Diego (M, 17, NZE) said: “I still only just do it socially. In the three years I’ve been vaping, I’ve never owned one… I just [do it to] have a bit of fun... not letting it get addictive… I’ve tried to keep it under control, not let it affect me too much....” People who intermittently owned vapes preferred disposable products, which they found cost-effective and easy to use; they associated reusable (ie, refillable) vapes with more serious, dependent users. Monica (F, 16, Asian) noted: “I don’t see myself getting a proper vape, ‘cause then it’s like admitting… it’s commitment.”

However, sharing brought new social pressures as norms of reciprocity and generosity evolved: Zack (M, 17, NZE) noted: “I feel like they don’t wanna be, like, that one person that doesn’t pass around *[to others in a group]*, ‘cause I think it’s so common now.” A small minority felt concerned sharing could spread disease and managed this risk by sharing only with people they knew. More complex dilemmas arose when younger students approached participants and asked to use their vape. Charlotte (F, 17, NZE) explained: “… if you say ‘no’, they’ll find something worse, and if you say ‘yes’, they shouldn’t be [vaping] anyway. So the way to go about handling it is quite hard.”

Sharing enabled participants to bond with their peers and bridge social groups. Those who did not own a vape depended on sharing others’ devices, yet maintained a privileged identity as nonaddicted. However, sharing posed challenges as device owners had to decide how they could vape responsibly.

### Social Purchasing: Developing a Regular Supply Route

Many participants made purchases via “proxies,” typically older friends or siblings aged 18+, who either bought products in-person from vape retailers or, in a minority of cases, from online stores. Once participants had an established supplier, they tended to rely on this proxy, who would often supply a wider group. Sally (F, 16, Māori) explained: “There’s always that one person who has… an older sibling or someone that’s fine with buying for them. And then everybody will ask them… whenever they want some.”

However, not all participants used a known proxy; a minority had developed online connections (via social media channels) with “randoms” (people outside their social circle) who sourced vapes for them. Charlotte (F, 17, NZE) explained: “…any random person who’s over 18… normally if they’ve got their Snap Map on *[a function that locates users on a virtual map]* and they’re in [the area], I’d just message them… either that or I start Snapchatting them the day before… to get to know them... Once I’m comfortable I’d pop the question… ‘Can you go to the vape shop?’… I either meet them or [usually] they drop it off at my friend’s house and I pick it up… It’s kind of like a drug deal.” Some participants had seen youth approach people outside a store, who they asked to buy a vape for them. Charlotte (F, 17, NZE) noted: “It’s the same with alcohol… you’d stand outside the shop, wait for someone to come out and just ask.”

Some participants had purchased vapes after seeing these advertised on social media: Zack (M, 17, NZE) explained: “On Snapchat, [people] just [post] on their story, saying [they’re] selling... People will just pop up, saying, ‘I’m keen to buy.’” Although these approaches avoided the need to recruit a proxy, they were opportunistic, did not create regular supply channels, and brought new risks. Not all online purchases would arrive, and those that did could contain damaged or used products; Fleur (F, 17, NZE) commented: “There’s a lot of scamming… people who are young and just got on nicotine, they’re vulnerable, they’ll pay the extra money and sometimes they won’t get what they paid for.”

Social purchase channels typically involved proxy purchasers who ranged from known and reliable agents to “randoms” identified on social media. Other proxies operated commercially and charged a fee for supplying vaping products; we examine this route in the section below.

### Quasi-Commercial Supply: Opportunities for Entrepreneurship

Where purchasers did not have a personal connection to their proxy, exchanges tended to be more commercial. Some proxies charged an additional sum (typically $5–$10) above the product cost as compensation for their efforts. Anton (M, 16, OE) noted: “…there’s that one 18-year-old at school always doing it, he just charges $5 more than what a vape costs... that’s why they come to him, because they know he’ll be available… no questions asked.” Entrepreneurs found opportunities to profit from younger adolescents, who were often desperate to access vapes. Maia (F, 17, NZE) explained: “…13, 14 year olds… I follow a lot of them on social media and I’ll see them post on their story, ‘Will anyone buy me a vape? I’m so desperate. I’ll give you twice what it’s worth.’” A minority had sourced vaping products from student dealers who operated within their school; Jennifer (F, 16, OE) commented: “There’s quite an economy running here… the [most] popular is alcohol and marijuana, or vape juice… I get juice [refills] from my person who sells me marijuana.”

Commercially oriented supply operations extended beyond business-minded individuals; participants also described networks that operated on alternative social platforms, such as Discord (an app traditionally popular among online gamers, with servers that are primarily private spaces accessible by invitation).^37^ These networks evolved through word of mouth, were managed by administrators who regularly disbanded groups to avoid detection, and sold a full range of products. Zoe (F, 16, Māori) explained: “… you send somebody a link and they join the chat… they have constant exposure [and] access to vapes and accessories. Whoever is selling… they’ll post an ad... [They’ll] have eftpos available, [or] cash. You comment under that photo and you’re done… [with] the option of picking up or getting it dropped off… after two days, we’ll delete them and add… a new lot of people into a new chat… the main dealers will change… there’s not much trace online when you are constantly refreshing and making new ones.” However, although participants knew of these supply routes, fewer had used them as the cost and complexity was less appealing than using peer or close suppliers.

### Commercial Self-Purchasing: Trying One’s Luck at Certain Retailers

Nearly all participants knew of commercial retailers who sold to underage youth; many reported purchasing vaping products from a “dodgy dairy” that did not require them to present an ID, and many knew peers who had fake IDs. Selma (F, 17, NZE) said: “There’s certain dairies that people always know of… small ones… [by] word of mouth. Someone would go in and not get ID’d and then tell people.” Some had learned store routines to facilitate successful purchases, visiting in the evening (when staff numbers were reduced) and approaching staff known to have a lax approach to age verification. Sally (F, 16, Māori) noted: “Some people told [my friend] that one of the workers there doesn’t care…” Many knew of younger adolescents who had purchased in person. Charlotte (F, 17, NZE) explained: “There’s so many kids who do that. I reckon that’s how the 13 and 14 year-olds get it, because they wouldn’t be friends with 18 year-olds. They just try their luck…”

Few participants had purchased from online retailers and several felt their parents could intercept online purchases or worried the delivery company would ask them for ID. Those who did purchase online arranged deliveries to older friends or used stores known to ship products in unmarked boxes or amongst other products, such as lollies. Amy (F, 17, Indian) explained: “I get someone who’s 18+ to order it, people from work… they send it to their house and then I go and get it.”

## Discussion

Our findings offer novel insights into the strategies underage youth use to access ECs (“vapes”). Nearly all participants accessed these products via social sources (mainly by sharing with peers); sharing was casual, normative, and rampant, although some felt troubled by requests to share with younger adolescents. Many used proxies, either known through social connections or “randoms” identified on social media, to purchase vapes for them. Quasi-commercial networks existed within schools and on social media, and some participants purchased in their own right, usually from smaller retail outlets.

We extend earlier studies, including a recent scoping review, that found social sourcing (eg, via personal contacts) to be the most prevalent EC access route among underage youth in several high-income countries, by exploring interaction dynamics in more detail.^27,28,38–40^ For example, we consider the parties involved in sharing and proxy purchasing, and the nature of these exchanges.

Sharing (or “passing around”) vapes, typically with close friends at parties or gatherings, strengthened friendships and feelings of belonging to a social circle, and created “bonding” social capital.^41^ By sharing with those outside their social circle, participants gained entry to new peer groups and generated “bridging” social capital.^41^ Sharing enabled some participants to maintain a valued persona: the nonaddicted user who remained in control and did not need to own a device; sharing thus symbolized both social use and inclusion. Just as “social” smoking allowed young people to distance themselves from the tainted identity of “smoker,” social vaping provided connections while maintaining a privileged status.^42^

Our findings identify current policies that have failed to protect young people. Participants had no difficulty identifying “dodgy dairies” and several sourced vaping products from commercial retailers known to waive age verification. Compliance, monitoring, and enforcement all require improvement, and policy makers could consider introducing escalating fines and other retailer penalties.^26,34^ A recent scoping review also found that underage commercial purchasing of vaping products (mainly from brick-and-mortar shops) remains a problem in several high-income countries where these sales are illegal.^40^

Upstream policy measures to reduce vaping product availability appear crucial to disrupting social supply channels (including sharing). Aotearoa NZ has proposed restricting new specialist vape stores from operating within 300 m of schools, but has not restricted existing specialist stores or generic retailers within this boundary.^43^ Stronger policy limiting product sales to stand-alone R18 specialist retailers (ie, disallowing sales in generic shops, including dairies and convenience stores) could more effectively reduce youth access and exposure. Capping vape retailer numbers could reduce density problems, decrease young people’s exposure to vaping products, and make these less accessible.

Aotearoa NZ also requires disposable vapes, the products most young people use initially,^44^ to have removable batteries^43^; however, new products are already circumventing this measure and these products appear likely to remain widely available.^45^ Alternative measures could include bans, currently under consideration elsewhere,^46^ excise taxes, minimum prices, and plain packaging, which could reduce these products’ affordability and appeal.

New policy measures should also address social supply via online channels, though a minority of our participants reported making online purchases. While social media platforms such as Snapchat prohibit (or limit) tobacco and vaping product sales,^47^ these policies do not appear to be adequately enforced. In addition, despite current advertising and sponsorship regulations, many would be exposed to commercial and user-generated vaping material online.^47,48^ Measures such as “age-gating” (restricting access to tobacco product sales and promotions unless age-verified accounts are used^47^) could help to reduce access. Furthermore, social media platforms could be more proactive in disallowing commercial and user-generated content.^47^

Our study has limitations. Multiple nonvalid responses delayed recruitment for Sample 1 and the limited pool of 16- to 17-year-olds living in university residential colleges meant we could not sample purposively to foster diversity. Nonetheless, the addition of the AFL 2.0 study data increased sample diversity and data richness, and helped contextualize identified themes, though our overall sample remains skewed toward female participants.

The sampling strategies for the two data sources varied, with one using online individual interviews while the second used in-person dyad interviews, followed by in-person interviews. This latter approach, involving two separate interviews, may have created a stronger rapport with participants. However, both studies prioritized rapport-building by involving interviewers of diverse ages and backgrounds, and allowing participants to shape the interview direction. We acknowledge that, as public health researchers, our perspectives and experiences differ from those of our participants; we reflected on these differences during regular team meetings while collecting data, and while interpreting and writing up the findings.

One interviewer identified as Māori and one as Pacific; however, for logistical reasons, not all Māori and Pacific participants in our overall sample were interviewed by Māori and Pacific researchers, respectively. As a predominantly Pākehā research team experiencing privileges and advantages not accessible to all, we may have missed culturally specific nuances relevant to the access and maintenance of EC/vape use.^49^

Because we aimed to probe supply route operation, an underresearched topic, we did not aim to complete separate analyses by ethnicity. However, given the varied social supply routes identified, and the high levels of vaping among Māori and Pacific adolescents,^50^ future work led by Māori and Pacific researchers should explore how young Māori and Pacific people access vaping products (see Supplementary File 5 for information regarding use prevalence by ethnicity). Future research could also explore experiences of young people living in rural and high-deprivation communities, who may evolve different access routes. In addition, future work could explore participants’ co-use practices, particularly vaping alongside alcohol consumption, and the integration of nicotine vaping with vaping of other substances, such as cannabis.

In summary, we have undertaken the first in-depth exploration of how underage youth access ECs in Aotearoa NZ, and extended earlier surveys that documented access routes but did not explain in sufficient depth how these establish, evolve, or operate. Our findings illustrate the highly varied ways in which young people access nicotine vaping products, offer new insights into how supply routes function, and expose serious gaps in current policy.

## Supplementary Material

Supplementary material is available at *Nicotine and Tobacco Research* online.

ntae096_suppl_Supplementary_Data_S1

ntae096_suppl_Supplementary_Data_S2

ntae096_suppl_Supplementary_Data_S3

ntae096_suppl_Supplementary_Data_S4

ntae096_suppl_Supplementary_Data_S5

## Data Availability

Data underlying this article will be shared on reasonable request to the corresponding author.
