# Supplementary material for: A Qualitative Analysis of How Underage Adolescents Access Nicotine Vaping Products in Aotearoa New Zealand
Source: Nicotine Tob Res. 2024 Apr 20;26(10):1370–6. doi: 10.1093/ntr/ntae096 (PMC11417153; doi:10.1093/ntr/ntae096)
Supplement: ntae096_suppl_Supplementary_Data_S1 [file ntae096_suppl_supplementary_data_s1.pdf]

qualtrics XM

Supplementary File 1: Online Eligibility Survey (Sample One)

Youth Vaping Recruitment

Start of Block: Background

Q1.1
Thank you very much for your interest in our study.

Before we get in touch with you to send you more information about the study, we would like you to complete a few short questions.

We would like to recruit diverse participants and your answers will help us create a sample of people with different behaviours and characteristics.

If you have any questions, please contact me at janet.hoek@otago.ac.nz, or phone 03 479 7692

Page Break

Q1.2 **Vaping Background How old were you when you FIRST HAD A PUFF on a vape?**

____

Page Break

qualtrics XM

Q1.3 **What sort of vape have you EVER used?**
Please tick all that apply

- ☐ **A disposable e-cigarette that cannot be recharged (sometimes called a cig-a-like):** (1)
- ☐ **An e-cigarette that has replaceable cartridges (sometimes called a vape pen):** (2)
- ☐ **A rechargeable e-cigarette that has a tank you fill with e-liquid (sometimes called a 'mod' or 'tank'):** (3)
- ☐ **A refillable pod vape (sometimes called a pod mod; examples are Juul or Vuse)** (9)
- ☐ **A disposable vape that has a set number of puffs and is thrown away when finished (examples are Puff Bar or Elf Bar)** (10)

Page Break

Q1.4 **Do you own your own vape?**

- ○ Yes (1)
- ○ No (2)

Page Break

Q1.5 **IN THE PAST 30 DAYS, have you used a vape?**

- ○ Yes (1)
- ○ No (2)

qualtrics XM

End of Block: Background

Start of Block: Ineligible

Q2.1

Thank you for your interest in our study, unfortunately you are not eligible to participate.

End of Block: Ineligible

Start of Block: Vape used last month

Q3.1 **IN THE LAST MONTH (i.e., the last 30 days), on how many days did you use a vape?**
Please click on the number in the dropdown box

▼ 1 (1) ... 30 (30)

Page Break

Q3.2 **IN THE PAST 7 DAYS, what type or types of vape have you used?**
Please tick all that apply

- ☐ **A disposable e-cigarette that cannot be recharged (sometimes called a cig-a-like):** (1)
- ☐ **An e-cigarette that has replaceable cartridges (sometimes called a vape pen):** (2)
- ☐ **A rechargeable e-cigarette that has a tank you fill with e-liquid (sometimes called a 'mod' or 'tank'):** (3)
- ☐ **A refillable pod vape (sometimes called a pod mod; examples are Juul or Vuse)** (9)
- ☐ **A disposable vape that has a set number of puffs and is thrown away when finished (examples are Puff Bar or Elf Bar)** (10)
- ☐ **Other:** (4) ____

End of Block: Vape used last month

Start of Block: About Yourself

Q4.1 **About Yourself How old are you?**

____

Q4.2 **Which ethnic group or groups do you belong to?**
Please tick all that apply

- ☐ New Zealand European (1)
- ☐ Māori (2)
- ☐ Samoan (3)
- ☐ Cook Island Māori (4)
- ☐ Tongan (5)
- ☐ Niuean (6)
- ☐ Chinese (7)
- ☐ Indian (8)
- ☐ Other European (9)
- ☐ Other (such as Fijian, Korean) (10)
- ☐ Other (please specify) (11) ____

Q4.3 **What is your name?**
Just a reminder that we will not share any information about you, or your answers with anyone outside our research team

____

---

qualtrics XM

Q4.4 **How would you prefer us to get in touch with you?**

- ◯ Mobile only (1)
- ◯ Email only (2)
- ◯ Either mobile or email is fine (3)

Display This Question:

If How would you prefer us to get in touch with you? = Mobile only

Or How would you prefer us to get in touch with you? = Either mobile or email is fine

Q4.5 **What is your mobile number?**

____

Page Break

Display This Question:

If How would you prefer us to get in touch with you? = Email only

Or How would you prefer us to get in touch with you? = Either mobile or email is fine

Q4.6 **What is your email address?**

____

Page Break

qualtrics XM

Q4.7

Thank you for your help with our research
If you are eligible to take part in our study, one of the research team will be in touch with you soon

Please click NEXT to submit your questionaire

End of Block: About Yourself
