# Supplementary material for: A Qualitative Analysis of How Underage Adolescents Access Nicotine Vaping Products in Aotearoa New Zealand
Source: Nicotine Tob Res. 2024 Apr 20;26(10):1370–6. doi: 10.1093/ntr/ntae096 (PMC11417153; doi:10.1093/ntr/ntae096)
Supplement: ntae096_suppl_Supplementary_Data_S2 [file ntae096_suppl_supplementary_data_s2.pdf]

<u>**Supplementary File 2: Participant Flowcharts and Recruitment Details**</u>

Sample 1

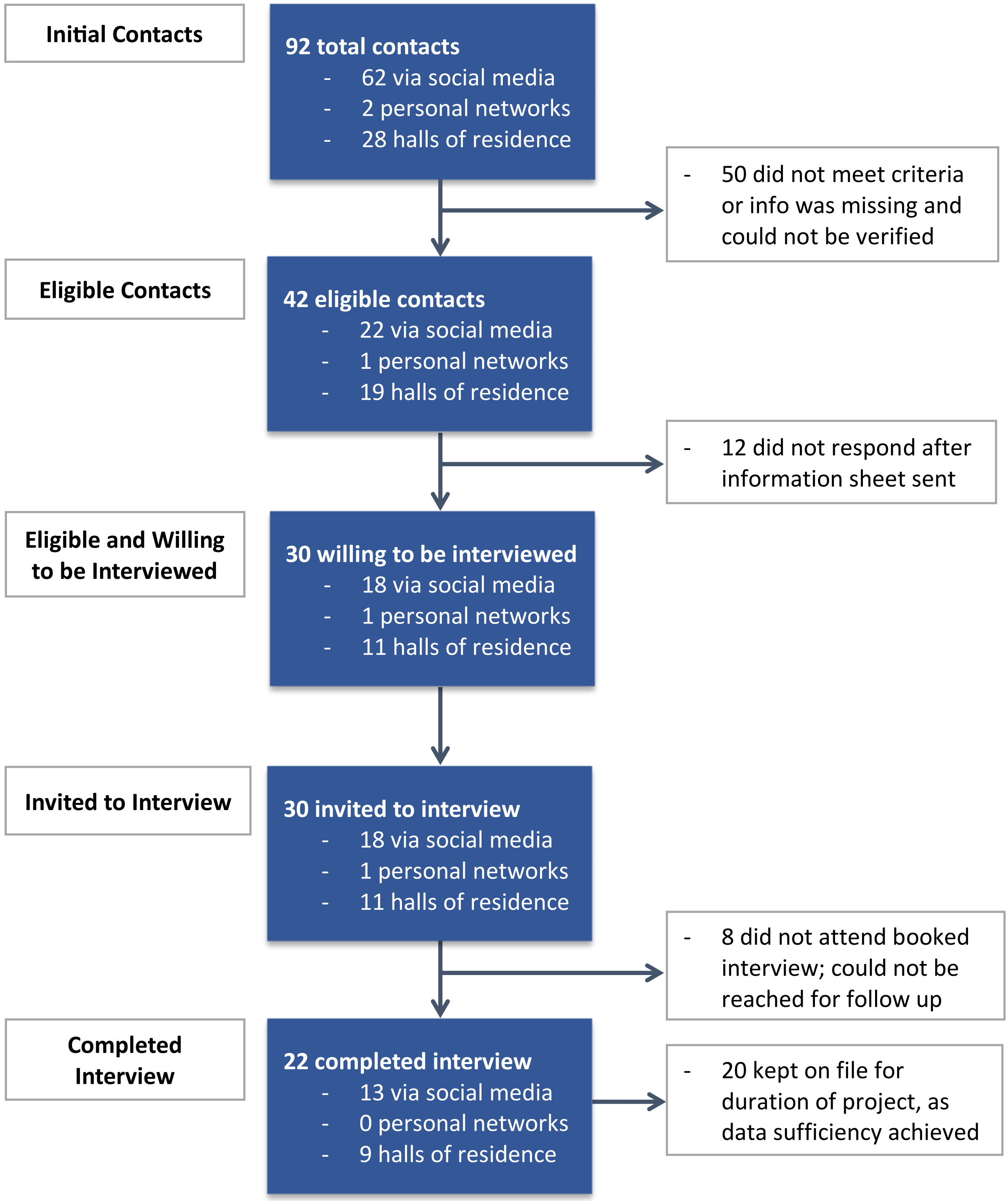

Sample 2*

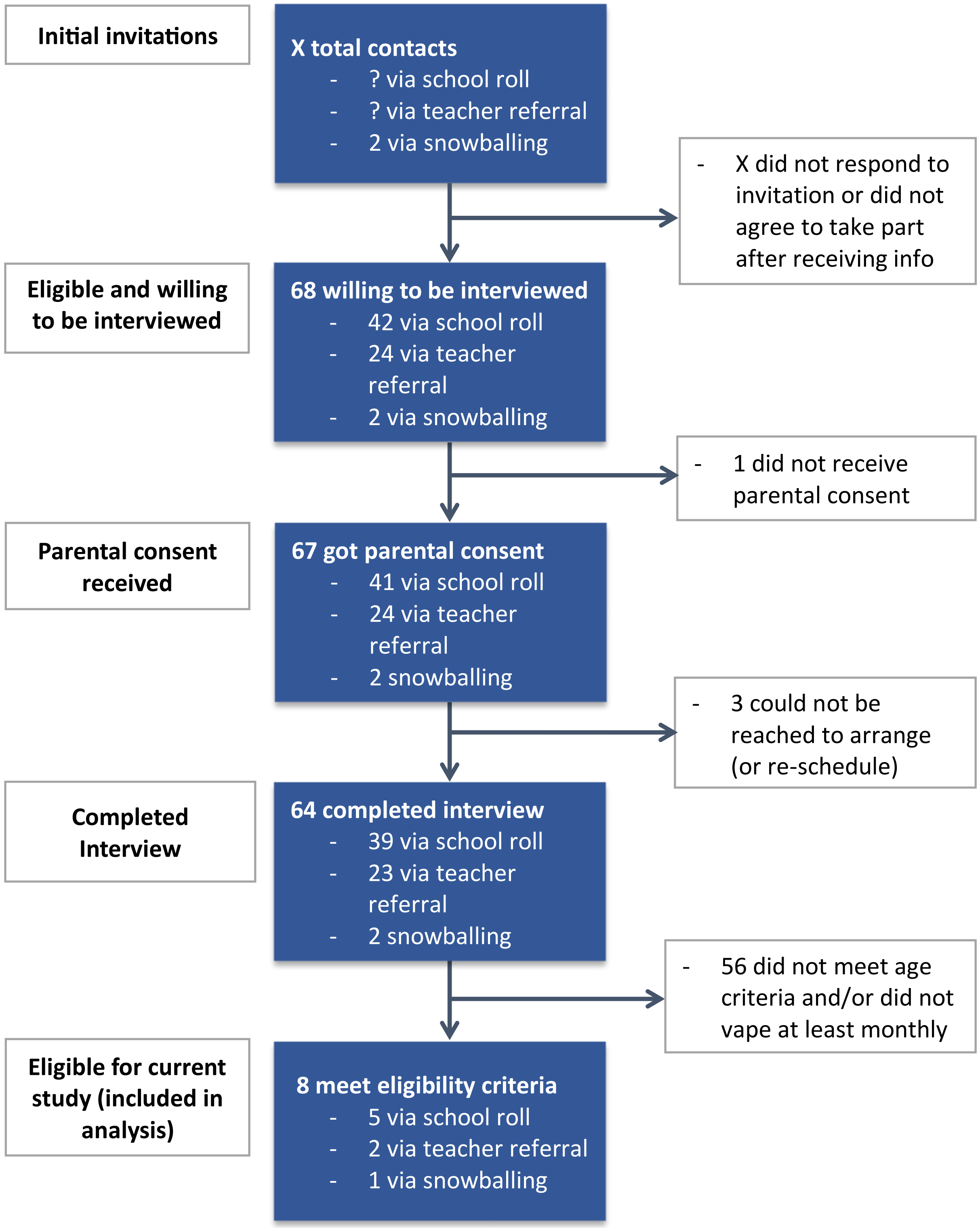

* Insufficient data to fill in the top most layer. The response rate to the initial invitation (a letter delivered by homeroom teachers) was low overall (~1 in 5 attended the study briefing) and even lower for Māori students and boys. It is unlikely that all invitations were delivered, and some invitees turned out not to be attending school any longer, making it difficult to estimate the true response rate. The response rate for the teacher referral process was much better (~50% - 75%) but it remains unclear exactly how many were invited. Nearly all who came to the study briefings agreed to participate.

Social Media Advertising Poster (Sample One)

UNIVERSITY of OTAGO
Te Whare Wānanga o Otāgo
NEW ZEALAND

Exploring vaping among NZ youth

- **Are you aged 16-17 and vaping at least once a month?**

We want to hear your thoughts!

Project Description

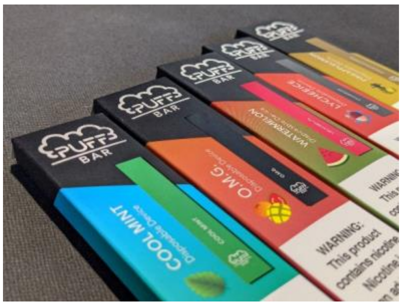

- We're keen to kōrero with people aged 16 or 17 who have vaped in the last 30 days.
- We can arrange an interview with you by yourself or you can bring a friend aged 16-17 who has also vaped in the last 30 days to come with you.
- The interview will take around 60 minutes; we'll arrange a time that suits you.
- **Your participation will be strictly confidential**.
- If you would like to take part in this project, **please click on the link in the FB post** and complete a short survey to check you are eligible to take part.

We will offer you (and your friend) a koha ($30 Warehouse voucher per person) to acknowledge any costs you might have from taking part in our study.

Eligibility

To participate in the study, you must:

- Be aged 16 or 17;
- Have vaped at least once in the last 30 days;
- Be able to participate in person or online (via Zoom)

Contact Details

If you are interested in participating or would like more information, please contact:
Janet Hoek, janet.hoek@otago.ac.nz
Katie Frost froka813@student.otago.ac.nz

This study has been reviewed and approved by a Departmental Advisor on behalf of the University of Otago Human Ethics Committee.

Recruitment Invitation to Year 12 (High-School) Students (Sample Two)

Adolescent Friendships and Lifestyles 2.0

Kia ora, Talofa lava, Kia orana, Mālō e lelei, Mālo ni, Fakaalofa atu, Kumusta, 你好

YOU ARE INVITED TO PARTICIPATE IN A RESEARCH PROJECT

Hi

We would like to invite you to take part in a study called 'Adolescent Friendships and Lifestyles 2.0' (AFL2.0). You have been randomly selected from your year group to take part.

What is AFL2.0 about?

The AFL2.0 study aims to understand what it's like to be a young person today, and how the lifestyles and attitudes of young people have changed from 20 years ago. By getting a better understanding of young peoples' lives, the research will also inform local and national efforts to promote young people's wellbeing.

What will I have to do?

If you decide to take part, you will have two interviews – one with a friend, and one individual interview – each 25-45 minutes long. The interviews will generally be during school hours, but if you're concerned about missing class, we can schedule your interviews for a study period, lunch hour, or after school. At the end of the interviews, you will get a $20 voucher as a thank you for taking part.

Come to our information session to hear more – and bring a friend!

Please choose a friend who you would like to be interviewed with (and who is interested in being involved) and come meet the research team on **Tue 7 June at 10.35 (Form period), Library Annexe.** During this meeting we will tell you more about research project. You and your friend will have a chance to ask questions, along with others invited from your year group. At the end of the meeting, we will ask you to fill in a consent form, saying whether you want to take part or not.

What if I can't make it to the information session?

If you want to know more, but can't make it to the information session, please contact the research team (email: jude.ball@otago.ac.nz) and we'll send you an information sheet and consent form.

Do I have to take part?

No, it's up to you. We encourage you to come along and hear more about the study before you decide. If you don't want to be involved, you can email jude.ball@otago.ac.nz and just write 'I want to opt out'. You don't have to give a reason.

We hope to meet you soon!

Jude Ball (Pākeha)

Michaela Pettie (Ngāti Pukenga, Ngāti Maru)

Tatyana King-Finau (Tongan, Ngāti Ruanui, Ngāruahine)

AFL2.0 Research Team, University of Otago

Recruitment Criteria and Rationale for Age Limits

We report on two samples of eligible young people aged 16 or 17 who vaped at least once a month, lived in Aotearoa NZ, and could participate in an in-person or online interview exploring EC access. In Aotearoa NZ, parental permission is required to interview young people aged under 16; because many young people hide vaping from their families, we limited our sample to young people aged 16 to 17, who could consent to participate on their own behalf. We did not include those aged 18+ as these young people are of legal age to purchase vapes in Aotearoa NZ, and we aimed to probe underage use and access.

*Note for an international readership: In Aotearoa NZ, 18 year-olds may still be in high-school.

Non-Valid Responses During Recruitment (Sample 1)

JH and KF initially recruited 13 high-school participants using social media and personal networks. We treated all responses as valid and arranged interviews with people who met our eligibility criteria. However, we conducted two interviews that differed in several important respects from earlier interviews. First, participants refused to turn on their camera; second their responses suggested they lived outside Aotearoa NZ. We subsequently checked all IP addresses, which the Qualtrics survey platform captured, and excluded anyone whose IP address was not located within Aotearoa NZ. We excluded the two ineligible participants' transcripts from our analyses.

Surges in non-valid responses typically occurred after we had advertised for participants using social media, and significantly disrupted the recruitment process; adaptations were therefore required to reach those living in Aotearoa NZ. In total, we received 474 non-valid responses. Faced with these challenges, we modified our recruitment strategy. ADM approached University of Otago residential colleges as students arrived for the new academic year. Using this approach, she enrolled nine participants aged 16-17; as recruitment took place via college internal communications networks, we did not encounter non-valid responses.

Participants recruited through this approach had all very recently left high school (a couple of months prior) and had lived in halls of residence for no more than one month. We asked them to reflect retrospectively on their time in high-school when answering questions, so that we could understand how they were sourcing/accessing vaping products at a time when far fewer of their friends and peers would have been of legal purchase age (i.e., 18+ in Aotearoa New Zealand). The high school experience was important for us to parse out, because it represented a time when accessing vapes likely required different access routes and strategies for these participants. We used the same interview guide but made slight modifications to the framing of questions, to account for retrospective consideration.

School-Level Recruitment Details (Adolescent Friendships and Lifestyles- AFL 2.0 Project)

The eight eligible students included in the current analysis ('Sample Two' in our manuscript) were part of a larger study sample (AFL 2.0) focused on the function and meaning of substance use and non-use in adolescents' lives (see: www.otago.ac.nz/AFLstudy for details). Study organisers for AFL 2.0 did not formally advertise for this study; rather, they invited selected students to participate via a personalised letter (Pg. 4, above).

The reason this study used a selection process, rather than inviting all eligible students at the participating school, was because they aimed to achieve a diverse sample that represented the school community. A general invitation typically results in a self-selected sample and overrepresentation of conscientious 'student council' type students; a wider recruitment approach would require screening and then rejecting many who had volunteered to achieve a diverse sample, which the study team saw as ethically unacceptable.

AFL 2.0 aimed to recruit 60 participants aged 14-17 years, with at least 14 Māori and 14 Pacific students, so that stratified analysis by ethnicity was viable and Māori-specific and Pacific-specific research outputs could be prepared. The table below outlines recruitment aims. Researchers also

wanted to ensure the sample included students from different socioeconomic backgrounds, and students who might not typically volunteer to be interviewed.

The randomisation process was as follows. The school provided study researchers with the school ID number and ethnicity of all the students enrolled in Years 10, 11 and 12, in an Excel file. They stratified by ethnicity, randomised using Excel's randomisation function, and selected the appropriate number to be invited for each Year group/ethnicity. For each category they selected more students than needed (particularly for Māori students who they expected would be harder to recruit), to provide 'back up' students, if those initially chosen declined to take part or could not be contacted. They then worked with a school staff member, who looked up the student names corresponding with the ID numbers selected, and named the invitation letters and added names to their Excel file, so they could track who had responded and who had not.

The invitation letters were distributed via the students' form teachers, who had been briefed about the study. This approach resulted in a sample of 41 that was skewed towards girls and with Māori under-represented, as boys and Māori were less likely to take up the invitation to attend a briefing about the study. In the second phase of recruitment a purposive approach was taken, using snowballing and working with the deputy principal and deans to identify and invite students belonging to groups that were underrepresented.

The final sample involved 64 students: 30 male, 32 female, 2 non-binary; 18 Year 10s (14-15 years old), 26 Year 11s (15-16 years old), 20 Year 12s (16-17 years old). The participants were socioeconomically and ethnically diverse, including 16 students of Māori descent and 15 of Pacific descent. Eight of these 64 met the inclusion criteria for the current study re. vape product access. Eight of the 64 met the criteria for inclusion in the current study re. experiences of addiction

	Māori	Pacific	Non-Māori/non-Pacific	Total
School students				
Year 10 (14-15 years)	5	5	10	20
Year 11 (15-16 years)	5	5	10	20
Year 12 (16-17 years)	4	4	8	16
School leavers				4
TOTAL				60

Note re. Differences in Voucher (or Koha) Values Offered to Each Study Sample

In this study we report on two separate samples of eligible young people, who were recruited as part of two individual studies in different parts of Aotearoa NZ. We agreed on a combined data approach for this analysis, since each study explored underage EC access in depth. Each study team, however, had their own budget , goals and funding source. The study protocols were thus designed at different times and the koha (a gift to recognise participants' time) differed for these reasons.
