# Supplementary material for: A Qualitative Analysis of How Underage Adolescents Access Nicotine Vaping Products in Aotearoa New Zealand
Source: Nicotine Tob Res. 2024 Apr 20;26(10):1370–6. doi: 10.1093/ntr/ntae096 (PMC11417153; doi:10.1093/ntr/ntae096)
Supplement: ntae096_suppl_Supplementary_Data_S3 [file ntae096_suppl_supplementary_data_s3.pdf]

Supplementary File 3: Interview Guides

SAMPLE 1 INTERVIEW GUIDE

Exploring vaping among NZ youth

Kia ora, my name is Anna… I arranged to meet with you to talk about vaping and your experiences of vaping.
Tell the participant about myself and where I come from.

Offer to begin with karakia (Māori incantation or prayer)
Proposed karakia to open discussion:
Whakataka te hau ki te uru
Whakataka te hau ki te tonga
Kia mākinakina ki uta
Kia mātaratara ki tai
E hī ake ana te atakura
He tio, he huka, he hau hū
Tīhei mauri ora!

Is there anything you would like to tell me about yourself before the interview starts?

Before we start I'd like to show you some information about the interview and check to see whether you have any questions.

PROVIDE INFORMATION SHEET AND GO THROUGH IT WITH PARTICIPANT.

With your permission, we'll audio record this session and have it transcribed word-for-word USING AN ONLINE SERVICE CALLED REV.COM. Of course, everything you say will be anonymous (meaning, we'll give you a fake name). If you do not feel comfortable with me using this transcription service, please let me know and I will not use this function.

IS IT ALIGHT WITH YOU IF I TURN ON THE RECORDER NOW?

Thank you, I've now turned it on and I'll just read out some key points from the info sheet.
I would like to check that you are aged either 16 or 17? **[Participants to confirm]**
I'd also like to remind you of your rights as a participant in this project.

Key points about consent to read out while the recorder is turned on:

- As a participant in the research, you have the right to ask questions at any time; if there is something that is not clear, please tell me and I will do my best to answer your questions.

- You also have the right to decide if you would prefer not to answer some questions, so if we come to a topic you don't feel comfortable discussing, please let me know and we will move on.
- You have the right to stop the interview and withdraw from the study at any time **up until the point the interview ends**. If you decide to withdraw from the study, there is no penalty.
- Once I have your transcript, I will send it to you by email. If you have any comments on your transcript, you will be able to make these.
- If we use the automated transcribing and the software does not produce a high quality transcript, we will upload the audio file to Rev.com, which is based in the US and has people who transcribe from throughout the world. We would not know who would transcribe this interview but because some transcribers are based in New Zealand, there is a very small possibility that a transcriber may recognise your voice. We think the chances of you being recognised by a human transcriber are very, very small.
- We will use our best efforts to keep your responses confidential to members of the research team named on the Information Sheet.
- At the end of the interview I'll ask if you would like a copy of your interview transcript, summary of your interview, and summary of the overall research findings. I will also offer you a $30 Warehouse voucher.

DO YOU HAVE ANY QUESTIONS? PLEASE REMEMBER THAT YOU CAN ASK ME QUESTIONS AT ANY TIME DURING THE SESSION.

BEFORE WE GET STARTED, COULD YOU PLEASE SAY 'I CONSENT TO TAKE PART IN THIS PROJECT'?

Introduction

1. I'll start by asking you a bit about your first experiences with vaping. When did you first see or hear about vaping?
   - **Probe** information sources mentioned (seeing friends, family, social media, seeing product in store, seeing advertising or other marketing).
   - What did you think when you saw people vaping early on?
2. What got you thinking about trying a vape?
   - **Probe further re:** to quit smoking or factors leading non-smokers to start using; curiosity about experience and flavours, tricks, peer pressure, coolness of device, or presence of vaping in social settings.
3. Just thinking about your own vaping…
   - How long ago was it that you <u>first</u> tried a vape?
   - Where did you get it from? Who were you with and where were you when you tried it?
   - What sort of vape was it? (use visual images if needed)

4. Can you tell me what's happened with your vaping since you first started?
   - How often do you vape now?
   - **IF EVERY DAY**, ASK "How many times a day do you vape at the moment? Do you know more or less how many puffs you'd have each time you vape?"

   - Nowadays, what sort of vape would you use most often?

- **Probe further:** What makes you prefer that type? (e.g., cost considerations, ease of use the way it works), appearance, ability to vape covertly.

5. How much nicotine do you normally have in your vape?
   - **Probe:** Has that changed over time? How has it changed?

6. Overall, about how many pods (or refills) would you use in an average week?
   - About how long would A given pod or refill normally last you?
   - **NOTE:** IF VAPE IS DISPOSABLE, ask how many vapes they'd use in an average week.
   - About how long would a disposable vape normally last you?

7. What would you say is the main reason why you've continued to vape?
   - **Probe:** physical need to vape; psycho-social reasons (stress or anxiety relief); social reasons (popular thing among peers)

Vape devices

8. **FOR PEOPLE WHO OWN VAPES (**In the survey you did online, you said you own your own vape?)
   - How many vapes do you own?
   - If more than 1 vape owned: "Of the different vapes you have, what's your favourite?"

9. Just thinking about your vape (or your FAVORITE vape, if more than one owned), what brand/model is it? What do you like about it?
   - **Probe** attributes such as appearance/colour/size/features, ease of use, easy to hide, popular with peers.

So I'll move on to asking you some questions now about accessing or sourcing vapes…

10. Just thinking about the vapes you've owned, where did you get these from?
    - **Probe sources** – friends, social contacts, family, purchased

11. What's the main way you get vapes now?
    - **Probe sources** – friends, social contacts, family, purchased
    - **Probe:** What makes you go to [source mentioned] most often?

---

12. **FOR SOCIAL SOURCES**
    - You said you got given a vape from _____ (e.g., friends and/or family).
    - Can you tell me how you got a vape from _____? (e.g., did you ask for a vape?
    - Pay someone to get it for you? get offered by someone? (passed on from someone else or given a vape as a gift)
    - How often would you get a vape from _____?

13. Do you ever use someone else's vape/share with others?
    - About how often would you do that?
    - What makes you want to use someone else's vape/ what's the appeal there? (e.g., different design, flavour, they offered or it was being passed around, other reasons).

---

14. **FOR SOCIAL-COMMERCIAL SOURCES**

    You said you bought vapes from people you know socially (e.g., friends or peers at school):
    - Can you tell me how that works?

    IF bought from others (e.g., randoms):
    - <u>Who</u> do you buy from, and <u>how</u> do you know you can buy from them?

15. <u>Where</u> do you buy vapes from [sources mentioned]?
    - **Probe:** At school, outside school?

16. What made you decide to buy vapes from [sources mentioned] rather than going to a store or online?
    - **Probe:** got refused purchase at a store, more convenient, cheaper, brand preferred (other reasons).

---

17. **FOR COMMERCIAL SOURCES**
    - You said you had bought a vape yourself…
    - <u>Where</u> have you bought from?
        - **Probe:** dairy or convenience store, service station, supermarket, specialist vape store, variety store like a $2 shop, pharmacy, mall or shopping centre kiosk, online, or somewhere else?
    - <u>Why</u> did you choose this/these source(s)?
        - **Probe:** ease of purchase (no age ID), retailer happy to sell to them, convenience (near home or school), cost (devices cheaper), has brand they like, **probe other reasons.**

18. When you **get/got** a vape from [source mentioned] **do/did** you get asked for ID to check your age?
    - What did you do?
    - What happened?

19. Has anyone ever refused to sell you a vape?
    - What sort of store was it?
    - Can you tell me what happened?
    - What did you do when you couldn't buy a vape?

---

20. **IF DID NOT ALREADY TALK ABOUT SHARING/BORROWING:**

- Do you ever use someone else's vape/share with others?
- About how often would you do that?
- What makes you want to use someone else's vape/ what's the appeal there? (e.g., different design, flavour, they offered or it was being passed around, other reasons).

21. Overall, how easy do you think it is for people your age to get vapes?

- **Probe:** How would you compare getting vapes today compared to a year ago (easier, harder or no difference)?
- What makes you say it's easier / harder?

22. Do you think people your age have changed how they get vapes?

- **Probe:** How did people get vapes in the past?
- Where are they likely to get vapes now?
- What's led them to change how they get them?

These are all the questions I'd like to discuss with you.
Are there any things you would like to tell me that we haven't already discussed?
Do you have any questions for me?

If interview began with a karakia, close discussion with a karakia mutanga:

Unuhia, unuhia
Unuhia ki te uru tapu nui
Kia wātea, kia māmā, te ngākau, te tinana, te wairua i te ara takatā
Koia rā e Rongo, whakairia ake ki runga
Kia tina! TINA! Hui e! TĀIKI E!

Check how participants are feeling.

"Sometimes talking about vaping can lead people to think they would like to quit. I have some information on quitting vaping that I can give to you, if you are interested. Would you like a copy of this information?"

If in-person, complete gift voucher receipt form (obtain signature).

Let participant know we will send Warehouse voucher to their email.

Check if they'd like a copy of their interview transcript, summary notes from the interview, and summary of the overall research findings.

SAMPLE 2 INTERVIEW GUIDE

STUDENT FRIENDSHIP PAIR INTERVIEW GUIDE – SHORT VERSION

- Self intro, settling in, kai, karakia (if relevant)
- Any Qs about the project, or the process?
- Confidentiality reminder – we will keep what you say private
- Time limitations?
- **TURN ON RECORDER**

Topic	Question prompts
Your friendship	Tell me the story of how you know each other/became friends
Wider friend group	Interests, likes/dislikes, characteristics<br>What do you do together outside of school? (Including online)<br>Typical weekend
Other friend groups<br><br>'Coolness' hierarchy	What kind of people belong to each group?<br>Interests/activities<br>Are some groups 'cooler' than others?<br>Explore characteristics of 'cool' students & those of low status<br>How important is social media in the lives of people your age?
Personal perceptions about different substances and users:	Card sorting activity [cards with drug names: alcohol, cigarettes, vaping, weed/marijuana, cocaine, meth/P, etc}. First remove any drugs that students don't know and add any that are missing. Based on your views:<br>- Accessibility continuum: Very easy – impossible to get hold of<br>- Acceptability categorisation: Ok for people my age, OK for people who are older, Never OK.<br>- Discuss (what make one substance more/less acceptable than another)<br>- What are the associations you have with... (alcohol, vaping, smoking, weed)? What kind of person do you think of?
Wider perceptions about different substances and users:	Wider views at school –do others hold similar or different views?<br>Perceptions of users<br>How are students who DON'T use any substances viewed?<br>Differences between youth and adult perceptions<br>If 'safety' comes up, explore meaning & importance of safety
Place of alcohol etc in social life of teens	How important – if at all - is alcohol in the social life of people your age?<br>How about vaping? Smoking? Other drugs?
Anything else	That's all the questions I have. Anything you want to add?
About you<br><br>(If not asked already)	Age<br><br>Ethnicity<br><br>Anything else about your identity

- **TURN OFF RECORDER**
- Reminder to keep conversation private
- What happens next – follow up interview (one right now, if possible), voucher after that

- Pseudonym
- Debrief sheet
- Closing karakia (if relevant).

STUDENT INDIVIDUAL INTERVIEW GUIDE – SHORT VERSION

- Self intro, settling in, kai, karakia (if relevant)
- Any Qs about the project, or the process?
- Confidentiality reminder – we will keep what you say private
- Time limitations?
- **TURN ON RECORDER**

TOPIC	QUESTION PROMPTS
HOME/FAMILY LIFE	Tell me about your family and your home life<br>Languages spoken at home. Cultural connectedness<br>Parents' use of alcohol, smoking, vaping, drugs
PARENTAL STRICTNESS, BOUNDARY SETTING	Parents relaxed or strict about where you go, when you come home?<br>Parents views on vaping, drinking etc – similar or different to yours?<br>Do you do things behind your parents backs? Eg?
PARENTS VS FRIENDS	Any tension between parents' and friends' expectations?
PERSONAL EXPERIENCES OF SUBSTANCE USE<br><br><br>E-CIGARETTES	Ever used? **If not, why not?** Ever been offered? Would you try, if offered?<br>Story of **first use**. How it developed from there<br>**Current use** – how often, when, where. If stopped: when, why?<br>What do parents think?<br>Devices & e-liquids – What kind? **Where do you get it?**<br>Do different people prefer different brands/types?<br>**Why do you do it?** Benefits?<br>**Worries**/concerns/downsides? Ever tried to cut down?<br>Could you stop if you wanted to?<br>Will you be using in 5 years time? Why?/Why not?
SMOKING	As above.<br>Which did you try first – vaping or smoking**?**<br>Differences between smoking and vaping – which you do prefer? Why?
ALCOHOL	As above.<br>Type of alcohol preferred<br>Social meaning of types/brands<br>Story about last time you drank alcohol
MARIJUANA	As above<br>Mode of use – smoking, vaping, edibles…<br>Differences between alcohol & marijuana – which do you prefer? Why?
OTHER DRUGS	Are there any other drugs you've tried? Tell me about it. (Same questions as above)<br>Is there anything you'd like to try in the future? Why not tried yet?
BECOMING AN ADULT	Where are you at in the transition from child to adult?<br>What does it mean to be a child/teenager/adult?<br>What are you plans for the future?
ANYTHING ELSE?	Anything you want to add?

- **TURN OFF RECORDER**
- What happens next – voucher will be sent today
- Debrief sheet (if not already just given)
- Closing karakia (If relevant).
