# Supplementary material for: A Qualitative Analysis of How Underage Adolescents Access Nicotine Vaping Products in Aotearoa New Zealand
Source: Nicotine Tob Res. 2024 Apr 20;26(10):1370–6. doi: 10.1093/ntr/ntae096 (PMC11417153; doi:10.1093/ntr/ntae096)
Supplement: ntae096_suppl_Supplementary_Data_S4 [file ntae096_suppl_supplementary_data_s4.pdf]

Supplementary File 4: Codebook

**Evolution of vaping practices**	
Experimentation in social settings (trial occurring organically)	*Clara: "We were at a party… had a few drinks… I remember thinking, 'It's going around the circle and everyone's trying it.' I was like, 'Oh, okay'… The people around me in friend groups and social situations constantly have vapes."*<br><br>*Diego: "Oh, it [was] just… the weekend, at a party, I saw someone have a vape, just all my mates. And I was… 'Okay, yeah, I'll just try it."*<br><br>*Josh: "It's everyone [among the student body] … it's so popular now."*<br><br>*Isabel: "A couple of my friends had their own, so I [was] just giving it a try… from then onwards, it just kept happening… especially in social environment[s] around lots of people."*
Vaping to satisfy curiosity; affiliate with social groups; reduce fears of missing out	*Laura: "I think they're just going through that like weird stage where they'll just do anything to fit in, and anything to be cool."*<br><br>*Louisa: "It's social... just as you drink with your friends, people vape with their friends."*<br><br>*Maia: "Part of it was just a curiosity thing, but [it was] also watermelon flavoured… quite a lot of the people that I know vape because of the taste."*<br><br>*Selma: "So, all my friends started vaping. It was really common at parties and stuff… And I was like, 'Oh my God… 'This must be cool, super fun. What am I missing out on?'"*
Resistance to the idea that peer pressure prompted vaping	*Amy: "I was at my friend's house for a sleepover, and she had hers on the bed and she's like, 'Just do it once, it's not gonna matter,' or something, but that…[it] was, like, banter. It wasn't actually peer pressure. Um, and I said no, and then she went away. And I was like, "Hm, I wonder what really is in this that people like it so much."*

	Isabel: "Friends [who were] doing it were like, 'Oh yeah, give it a try'... Not peer pressure, but just that kind of stuff [people say] when you're in a group of friends…"
Appealing physical sensations and tricks with vape aerosol	*Laura: "Well, they were drinking, but I wasn't, 'cause I had to drive home. And he's like, "Here, this is how you feel drunk without drinking alcohol." So, I suppose it just felt like that. It just felt like I had a couple of drinks, and I was like a little bit dizzy."*<br><br>*Pania: "…I learned about vape tricks and then I got more so that I could keep on practising and then all of a sudden I couldn't go a day without vaping"*<br><br>*Sally: "A lot of like year 10s at my old school they loved head spins. They would drag it. There's like this thing called dragging and you keep going and going until it blinks."*
Nicotine dependence (link to stress and anxiety); loss of control over one's vaping	*Ben: "Just before the exams… cause' I was a bit stressed, I would go, 'Well, I'm feeling stressed… I have a vape to calm myself down'. And then I would feel a bit calm again… [but] when I got stressed out again, I'd go do it again. This whole cycle."*<br><br>*Keira: "I was going through a really rough patch at my school. That's why it became a big thing for me. Then just stress, it also just helps with stress."*<br><br>*Tatiana: "I'm going to paint you a little picture… I get up, I use it… I'm walking to work, I use it… on breaks, I use it… walking home, I use it. So, it's just when I'm doing anything really… I think I'd say to myself, 'You shouldn't have used it, because it's just really addictive.'"*<br><br>*Zoe: "I would, um, probably start off with a pod a week at the beginning. And then, um, it escalated to me being able to do a pod in three days and then a pod a day... I found with my friends that might suffer from mental health issues… I notice that their intake will increase if [they're] going through, perhaps… an episode of that….And I'll see their usage become more… harsh, harsher flavours…"*
Active monitoring to reign in one's use	*Diego: "Basically, I'm trying to limit myself, but whenever I see my mates… and they have one… yeah, I'd have them maybe 2-3 times a week now. I'll do it maybe a couple times a night, just to get the sensation. Other than that, no extensive chain vaping… just… keeping it chill and whatnot… Basically, I trust myself to make the right decisions in*

	life. And I know that obviously, vaping… isn't good for you. But I know if I keep it under control, then a bit here and there won't harm me."
**Sharing: a rampant and opportunistic social phenomenon**	
Highly common in social settings (parties, outings), especially among friend groups	*Clara: "If I'm in a social gathering and someone had their [vape] I'd be able to just say, "Can I get some? …It's definitely very, like, 'Yeah, sure.' They'll just give it to you... the attitude towards it, if there was someone who you didn't really know and you asked, 'Can I have some?' they'd be fine with it. I mean, on St. Patrick's Day, for example. There were so many people we didn't know around, but everyone was just casually sharing."*<br><br>*Diego: "Random people come up to you, and be like, 'Oh, hey mate, do you wanna vape?' Stuff like that… I have no clue who they are."*<br><br>*Isabel: "All your friends have [vapes] around you… it's definitely the social aspect of it… Just, all [my] friends are offering [to share]… anyone who's around you is open to offering to anyone [else]... [We share at] gatherings with friends… wherever we are… if we're all in a group and having a few drinks… that kind of stuff."*<br><br>*Kate: "Sharing them is very common… like, lots of people don't to want their own so they just share with friends..."*<br><br>*Keira: "You'll meet people at parties, and you've never seen them in your life, but you'll let them [share your vape]… also, if someone that's intimidating asks you, you're going to [share it with them]."*<br><br>*Mariana: "[Vapes are] being offered to me usually… by friends, sometimes my brother as well… [This happens] whenever I'm hanging out with them."*<br><br>*Sally: "If younger students do, the place that they will usually go to is the bathrooms …they usually go into the bathroom because there are a lot of girls in there that do it and then they'll just ask if they can have like a toke, and then a lot of people just give it to them. Even people that don't know each other."*<br><br>*Tatiana: "My sister and two best friends… whenever we hang out, we use each other's… it's just to try their flavour… you want to have theirs, you don't really think about it."*

Sharing reinforced identity as 'social' (non-committed) user; avoided the need to own a device	*Clara: "About six months ago I started vaping quite a lot more [a couple of times per week]... I started to think, 'Okay. This is probably a bit much.' I would find myself [wanting] it… I had a disposable for the New Year's period but, apart from that, I've never owned one myself… [Now, I vape in] social situations… it would still be a couple of times a week.'*<br><br>*Lizzy: "I did want to quit, so I was like, 'Okay. This is helping me quit.' It makes you feel a lot better if it's a disposable… being like, 'Oh, yeah. It's gonna run out. Then I'll just stop with this one and that'll be it.'"*<br><br>*Louisa: "I've never [owned] one… when you're at a party it's easier to just try someone's... just as you drink with friends, people vape with friends... Not many people have [reusable] pods anymore… it's mainly just dispos [that are being shared]."*
Sharing with randoms aroused concern re. disease spread	*Mariana: "I don't know what diseases they have in their mouth."*<br><br>*Selma: "Obviously, meningitis, COVID, all that kind of stuff. I don't want to get really, really sick and die or whatever just because I used some random person's vape."*
Sharing brought moral dilemma for some	*Zoe: "I would say the demographic of seniors we have now are just more on the mindset of distributing only to really their own ages and up… I feel like they have, they've seen how it's affected most of their own age and from when they started young. And I see that they don't want that from younger generations."*
**Social Purchasing: Developing a regular supply route**	
Common to access vapes via a proxy (often an older friend or sibling aged 18+ who could legally purchase)	*Diego: "If you're [underage]… you just get older people that you know, or a brother [to buy them]… stuff like that."*<br><br>*Maia: "16, 17 [year-olds]… most people would know at least one person who's over 18 who could [purchase for them]."*<br><br>*Rosie: "Junior students that do it, they usually are quite like confident and keen to do it …they just will message so many people until someone finally does it. Like… they'll just message everyone that they know that's older than 18 on their phone until someone says yes."*

Where no willing proxy available, online connections sought (with increased risk)	*Charlotte: "I'd say about 60 percent of them would say no [when asked to purchase via online channel]… the first person that says yes… I just stop [at that point]."*<br><br>*Pania: "One of my friends tried to buy a vape on snapchat… they never sent the vape… maybe if it was in person it could be a fair trade."*<br><br>*Rosie: "I have never done it online, but some of my friends have… usually it's rip-offs… it's broken, or just people trying to get a bit of extra money."*
Acquiring within high school peer groups	*Ben: "I bought it off of a friend… When people are selling their vape, they'll usually, uh, ask if anyone wants it… they'll just go around saying, 'Do you want to buy it?' …[Also], if you use someone else's vape and you say, 'Oh, that's good," then they might be like, 'Do you want it? I'll sell it to you.'"*
Approaching people randomly in public	*Keira: "I know people that will ask people that are just sitting outside the vape shop or stuff… will ask them to go in. But it just feels safer getting my sister to do it."*
**Quasi-commercial supply: opportunities for entrepreneurship**	
Additional charges imposed by proxies (where no personal connection involved); opportunities to profit from younger youth	*Fleur: "This girl who was year 10… she paid some ridiculous amount, like $80, to get maybe, like, four disposables."*<br><br>*Keira: "I've bought juice [refills] at school multiple times… It's people in my year [who] make profit from it... [they] have older [contacts who] get them juice… It's $25 [for a bottle of juice], but a refill uses the tiniest bit… They'll tell everyone they have $5 refills… half the time they don't even vape, but they'll make profit from it."*<br><br>*Zoe: "They're hustlers, entrepreneurs really. It's not uncommon for you to go to school and if you have a vape that you can fill up with a bottle of juice… one of my friends, what he'll do with his is he'll buy a bottle of, say, 500ml… that'll last him for one week of filling everyone's vapes up at school. He charges $2 for that, and his profit after that at the end of the week can be $300... that started when we were in year 10."*
**Commercial Self-Purchasing: trying one's luck at certain shops**	

Successful self-purchasing at small shops (mostly dairies known via word of mouth to have lax approaches to age verification)	*Charlotte: "Heaps of dairies sell to underage people… they don't even check ID."*<br><br>*Isabel: "They never really asked… I guess if you kind of looked of age, or looked older, then they just wanted the money…"*<br><br>*Maia: "Normally people would stay away from chain shops or supermarkets… [they] ID almost anyone."*<br><br>*Rosie: "I know of like three [stores] that people go into, and it's just from conversations that I've had... Some of my guy friends have fake IDs and go in themselves… people have gotten fake IDs for other purposes… [so] they use them."*<br><br>*Sally: "I've done it myself… the guy didn't ask for ID… in the dairy there's this little room and it's got [an] 18+ [sign]… I went in there and got two of them- one for me and one for my friend."*<br><br>*Tatiana: "They asked me once [at the vape shop I purchase from], but I said I didn't have it on me. They just said, 'Okay, you need to have it next time.' They never asked again and I still purchase there."*<br><br>*Zack: "Yeah… from experience, I've seen about like 12 year olds go in [and] walk out with vapes… like, no questions asked."*
Online self-purchasing (a less common access route; tried by few)	*Ben: "People usually do just real life…like, through friends and stuff, because they can trust that they'll get it."*<br><br>*Laura: "Not many kids that get it delivered to their house. I think, like, the kids that do… either their parents know, don't care, or are completely oblivious to it."*<br><br>*Rosie: "I heard that [you] could buy [online], and that the person hides it and makes it look like it's just treats... I think usually the packaging is quite discreet. Like it just comes in like a black mailing bag…"*
**Minor Codes not reported in manuscript**	
Gifting (receiving product from someone without charge)	*Ben: "Someone around or one of my friends will have some juice. Usually, they'll just give it to you for free, 'cause they have quite a big bottle."*

	Zoe: "They started buying it for me without me asking, and then they'd just given it like candy… [because] they were gifting me vapes [it was] pretty much perfect… it was coming in frequent enough to the point where I never had to ask."
Borrowing (holding onto someone's device for a period of time; not in their presence)	*Sally: "I remember I gave mine away for like a week because I didn't need. She looked like she was going through withdrawals so I just gave it to her."*
Co-ownership (purchasing with the aim of using back and forth)	*Amy: "I only know one group of people… It was a three person thing… they all chipped in only to buy the vape, and then each day, one of them would have it."*<br><br>*Anton: ""I paid half for it. Like, we split it for the weekday. So like on a Monday I'd have it, Tuesday he'd have it, Wednesday I'd have it. And then on the weekend, we usually spend the night at each other's houses anyway."*
