# Supplementary material for: A Qualitative Analysis of How Underage Adolescents Access Nicotine Vaping Products in Aotearoa New Zealand
Source: Nicotine Tob Res. 2024 Apr 20;26(10):1370–6. doi: 10.1093/ntr/ntae096 (PMC11417153; doi:10.1093/ntr/ntae096)
Supplement: ntae096_suppl_Supplementary_Data_S5 [file ntae096_suppl_supplementary_data_s5.pdf]

Supplementary File 5: EC Use Prevalence by Ethnicity (Aotearoa NZ)

There are no stats specific to **youth EC use by ethnicity** in the New Zealand Health Survey (NZHS), which we have referenced in our manuscript's introduction (the survey presents overall population prevalence by ethnicity but small sub-sample numbers prevented reporting of vaping prevalence by ethnicity among 15 to 17 year olds in 2022).

The most recent ASH Year 10 Snapshot Survey (reporting on 14-15 year-old boys and girls in Aotearoa NZ) highlights profound inequities in vaping prevalence among underage youth, with over twice as many Māori (the indigenous peoples of Aotearoa) youth having vaped in the past month as compared to youth of European descent.[1]

- ASH Y10 Stats (final pg. of report):
    - **Ever tried vaping**
      Total: 40.1%
      Māori: 61.4%
      Pacific: 45.5%
      European/Pākehā: 37.6%
      Asian: 16.4%

    - **Regular vaping (past month)**
      Total: 18.2%
      Māori: 33.8%
      Pacific: 19.8%
      European/Pākehā: 15.7%
      Asian: 4.6%

    - **Daily vaping**
      Total: 10.1%
      Māori: 21.7%
      Pacific: 11.1%
      European/Pākehā: 7.6%
      Asian: 2.3%

Graphical representation of EC use prevalence over time (among teenagers in Aotearoa NZ), as presented in a recent Public Health Communication Centre (PHCC) briefing (produced using data from the New Zealand Health Survey and ASHY10 Snapshot Survey):[2]

Figure 3: Current (at least monthly) and daily smoking and e-cigarette use among 15-17 year olds, 2011/12 to 2020/21

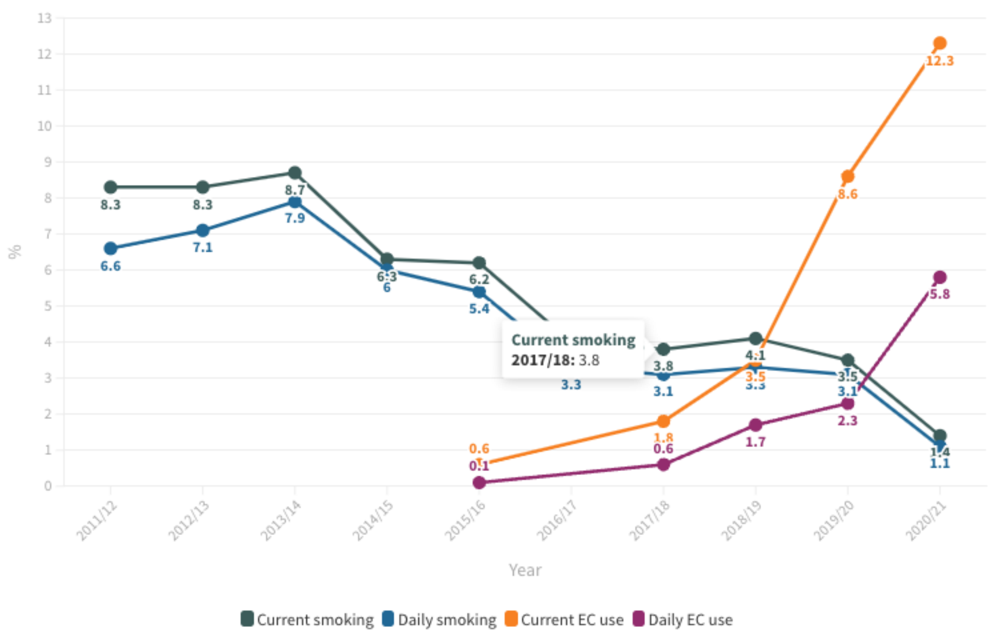

Source: New Zealand Health Survey Data Explorer • Current smokers defined as smoking at least monthly.

phcc

A Flourish chart

Figure 5: Daily vaping prevalence by ethnicity & gender (2015-2022) in ASH Year 10 Snapshot Survey

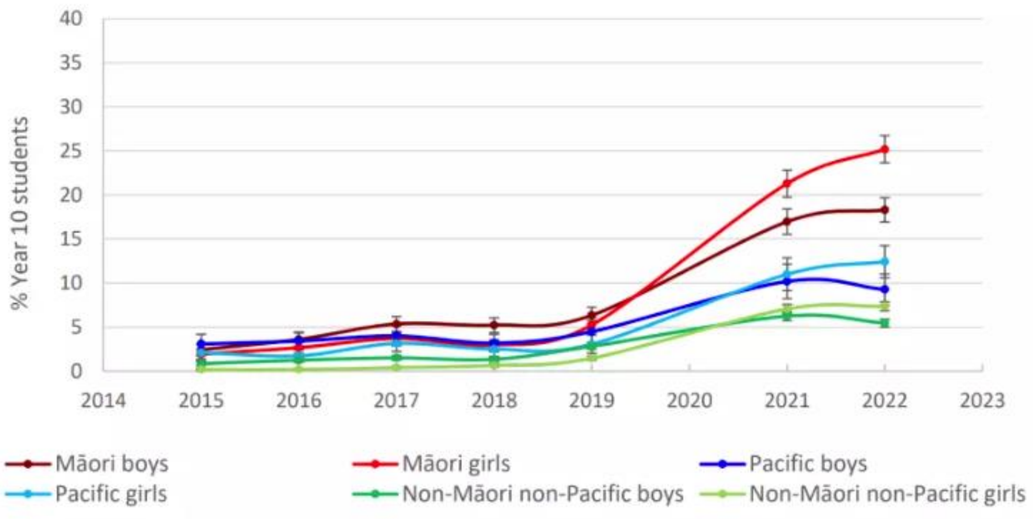

Source: ASH Snapshot Year 10 Survey[1]

References:

1. ASH (Action for Smokefree 2025). ASH Year 10 Snapshot Survey 2022: Youth Vaping and Smoking [Internet]. 2022. Available from: http://ash.org.nz

2. Edwards R, Hoek J, Waa A, Ball J. What is happening with vaping among adolescents and young adults in Aotearoa? Public Health Expert Briefing [Internet]. 2023 May 18 [cited 2023 Jul 7]; Available from: https://www.phcc.org.nz/briefing/what-happening-vaping-among-adolescents-and-young-adults-aotearoa

ASH Year 10 Snapshot Survey 2022

Topline – Youth Smoking and Vaping

The ASH Year 10 Snapshot surveys Year 10 students in Aotearoa New Zealand every year on their smoking and vaping behaviours and attitudes. It is one of the largest ongoing youth smoking surveys in the world, with 29,538 students participating in 2022. The Survey uses robust and validated measures and is conducted to a high methodological standard, that has been subject to peer review and ethics approval. It was not carried out in 2020 due to Covid-19. All results are shown with 95% confidence intervals.

Key findings

Daily smoking remains low and daily vaping increases only slightly; both regular smoking and regular vaping decrease; never smoking increases and ever tried vaping decreases

- Daily smoking rate remains low (1.1%); daily vaping rate increases slightly – there was a small but statistically significant increase (9.6% in 2021 to 10.1% in 2022). This was a much smaller increase than the previous few years
- Both regular smoking (4.2% to 3.0%) and regular vaping (20.2% to 18.2%) showed statistically significant decreases
- Never smoking rates showed a statistically significant increase (82.5% to 85.8%) and ever tried vaping rates showed a statistically significant decrease (42.7% to 40.1%).

Daily smoking rates by ethnicity and by ethnicity & gender remain the lowest since the Survey began; daily vaping increases and ever tried vaping decreases for Māori students

- Daily smoking rates by ethnicity and by ethnicity & gender remain the lowest since the Survey began
- Daily vaping increased a statistically significant amount for Māori participants (19.1% in 2021 to 21.7 in 2022), in particular for Māori girls (21.3% to 25.2%). There were no statistically significant changes in daily vaping rates for other groups when analysed by ethnicity and by ethnicity & gender
- Regular smoking decreased by a statistically significant amount for Māori (9.3% to 6.3%), Pacific (5.3% to 2.6%) and European/Pākehā participants (3.0% to 2.3%)
- Regular vaping decreased by a statistically significant amount for European/Pākehā participants (18.2% to 15.7%), but there was no significant change for other ethnicities
- Never smoking increased by a statistically significant amount for Māori (67.0% to 73.8%), Pacific (80.5% to 85.9%), and European/Pākehā participants (85.6% to 88.1). When also analysed by gender, this was the case for all groups except for Pacific boys
- Ever tried vaping decreased by a statistically significant amount for Māori (64.0% to 61.4%), Asian (19.0% to 16.4%), and European/Pākehā participants (40.3% to 37.6%).

Daily vaping among those who have never smoked increases slightly

- Daily vaping among those who have never smoked showed a small but statistically significant increase from 3.1% in 2021 to 4.3% in 2022
- There was no statistically significant change in daily vaping rates for those that also smoked daily (86.6% in 2022).

Daily smoking remains low and daily vaping increases slightly; never smoking increases and ever tried vaping decreases

Daily smoking rate remains low (1.1%); daily vaping rate increases slightly – there was a small but statistically significant increase in daily vaping (9.6% in 2021 to 10.1% in 2022). This was a much smaller increase than the previous few years. Daily use is those who answer that they vape or smoke at least once a day. See Figure 1.

The 2021/22 New Zealand Health Survey (NZHS) also showed an increase in daily vaping, which was accompanied by a decrease in daily smoking.[1]

Figure 1: Youth daily smoking (1999-2022) and daily vaping prevalence (2015-2022)

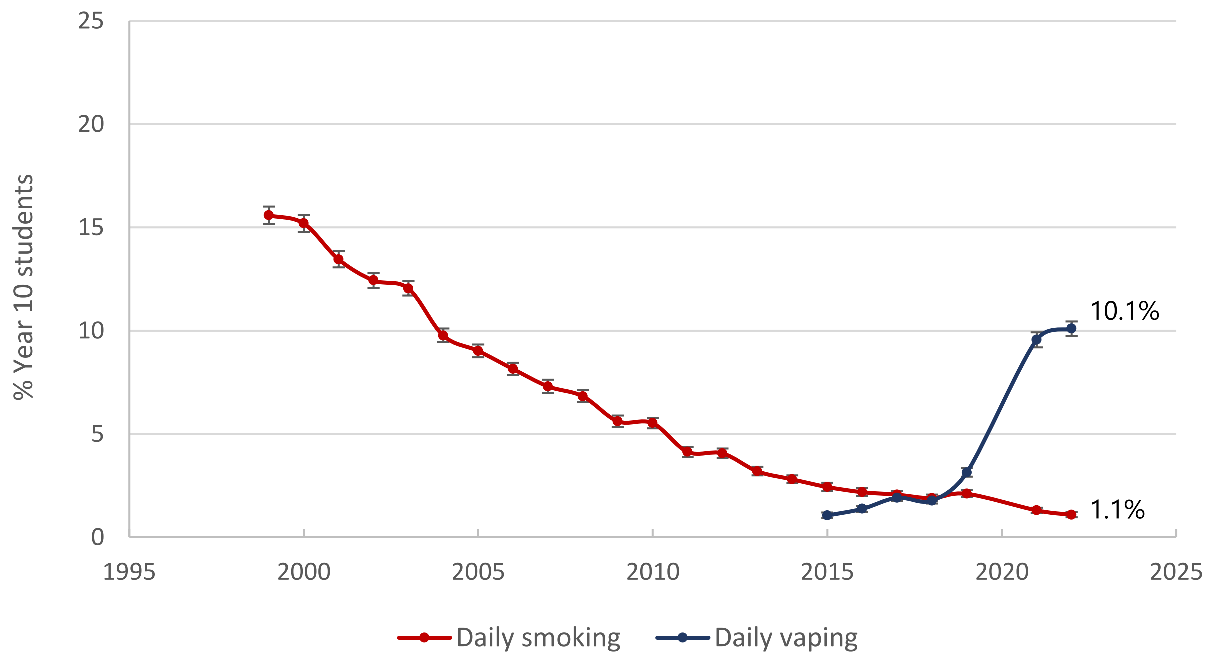

"Regular" use is defined as participants that report smoking or vaping either daily, weekly, or monthly. Both **regular smoking (4.2% in 2021 to 3.0% in 2022) and regular vaping (20.2% in 2021 to 18.2% 2022) showed statistically significant decreases**. This the first decrease in regular vaping rate that has been seen since the question was introduced. See Figure 2.

Figure 2: Regular smoking (1999-2022) and regular vaping prevalence (2014-2022)

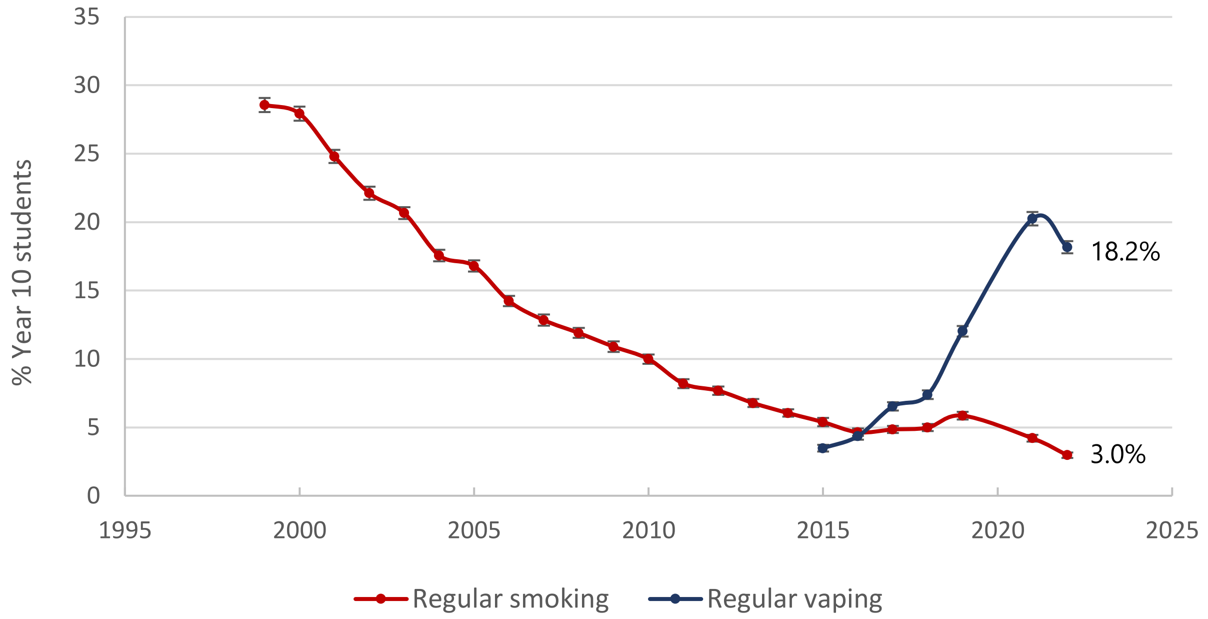

Never smoking rates measure the proportion of students who have never smoked a cigarette, even just a few puffs. **Never smoking increased by a statistically significant amount**, from 82.5% in 2021 to 85.8% in 2022 – the highest never smoking rate in the history of the Survey. See Figure 3.

"Ever tried" vaping is defined as having ever tried an e-cigarette/vape, even if it is just a few puffs or vapes. It is a measure of overall experimental use and includes both current vapers and one-off experimental use. **Ever tried vaping rates decreased by a statistically significant amount**, from 42.7% in 2021 to 40.1% in 2022. This is the first decrease in ever tried vaping that has been seen since the question was introduced. See Figure 3.

Figure 3: Never smoking (1999-2022) and ever tried vaping prevalence (2014-2022)

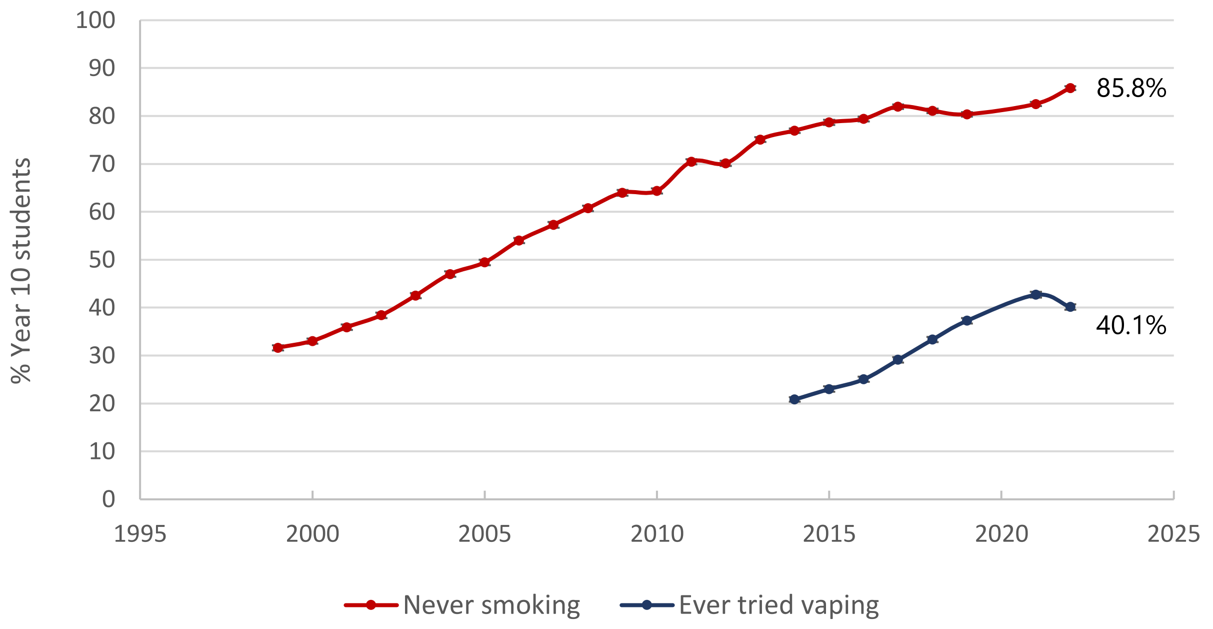

Daily smoking rates by ethnicity and by ethnicity & gender remain the lowest since the Survey began; daily vaping increases and ever tried vaping decreases for Māori students

Daily smoking rates by ethnicity and by ethnicity & gender remain the lowest since the Survey began, though there were no statistically significant changes between 2021 and 2022. See Figure 4 and 5.

Daily vaping increased a statistically significant amount for Māori participants (19.1% in 2021 to 21.7 in 2022), **in particular for Māori girls** (21.3% to 25.2%). There were no statistically significant changes in daily vaping rates for other groups when analysed by ethnicity and by ethnicity & gender. See Figure 6 and 7.

Figure 4: Daily smoking prevalence by ethnicity (1999-2022)

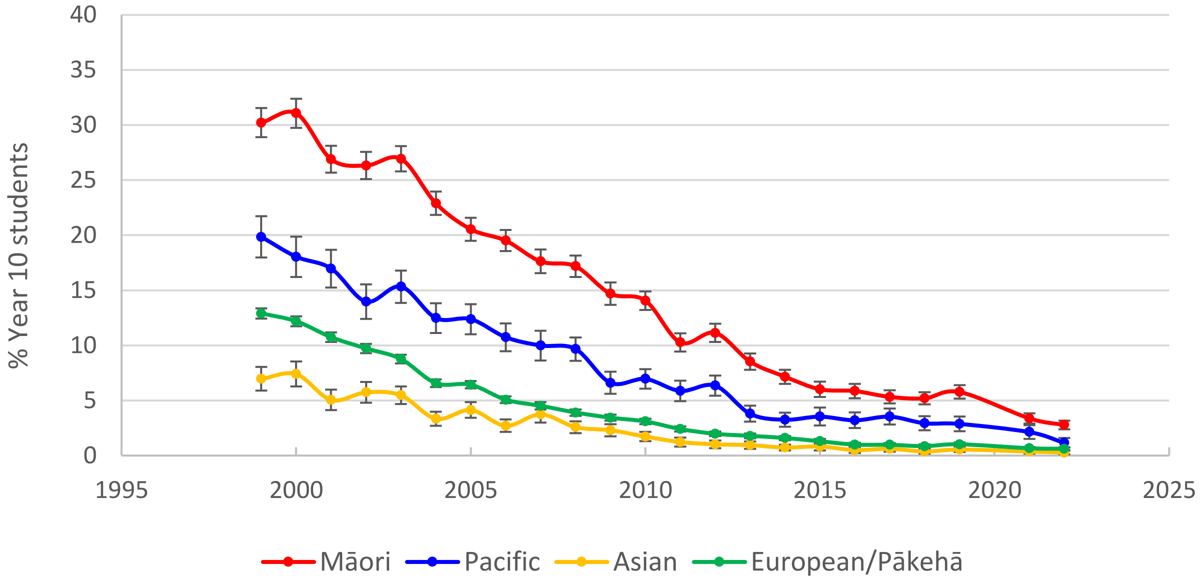

Figure 5: Daily smoking prevalence by ethnicity & gender (1999-2022)

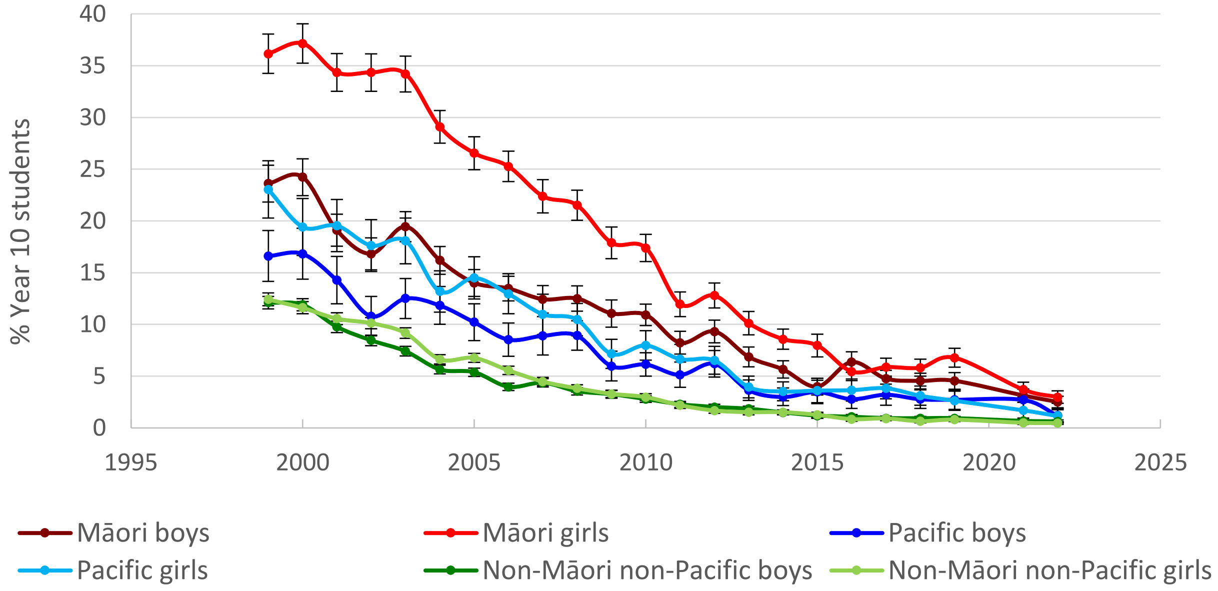

Figure 6: Daily vaping prevalence by ethnicity (2015-2022)

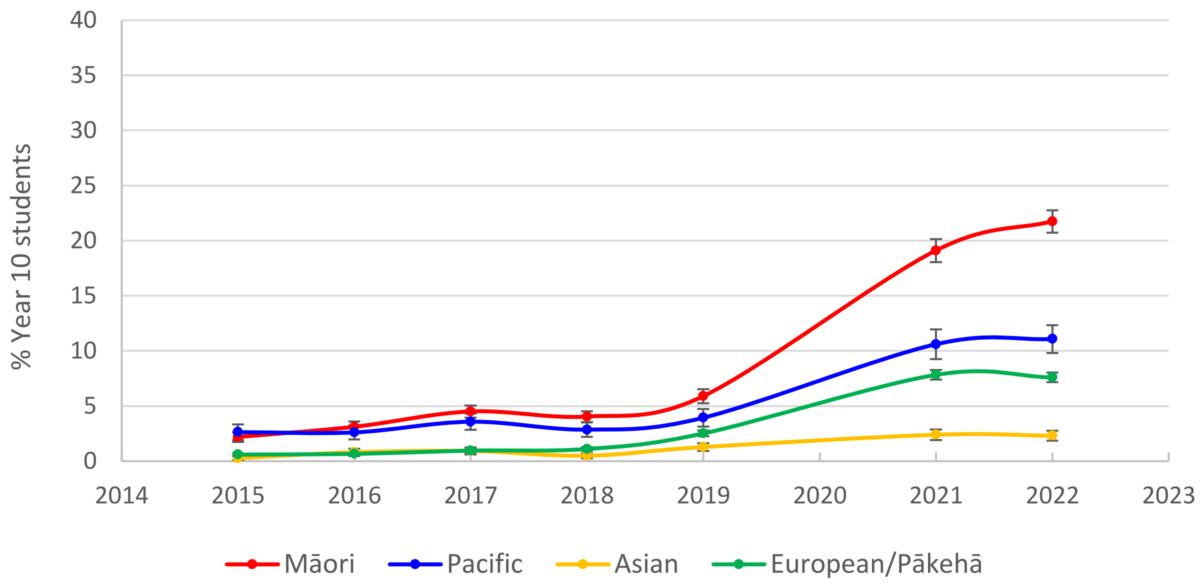

Figure 7: Daily vaping prevalence by ethnicity & gender (2015-2022)

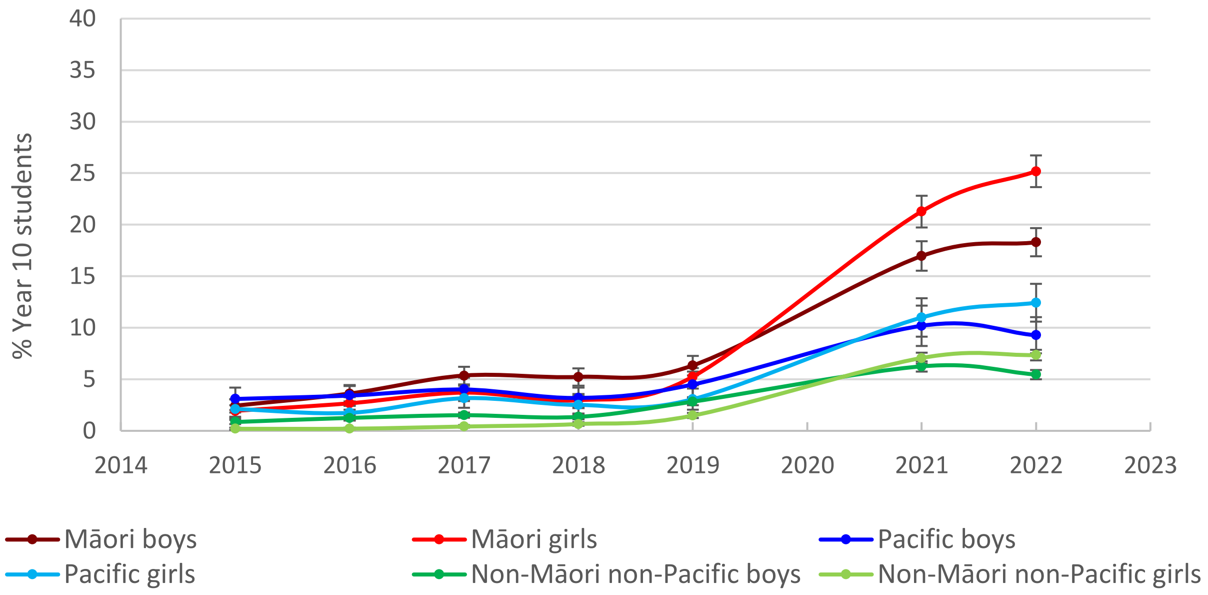

There was a statistically significant decrease in regular smoking rates for Māori (9.3% in 2021 to 6.3% in 2022), **Pacific** (5.3% to 2.6%) **and European/Pākehā participants** (3.0% to 2.3%). See Figure 8.

There was also **a statistically significant decrease in regular vaping rate for European/Pākehā participants** (18.2% to 15.7%), but there was no statistically significant change for other ethnicities. See Figure 9.

Figure 8: Regular smoking prevalence by ethnicity (1999-2022)

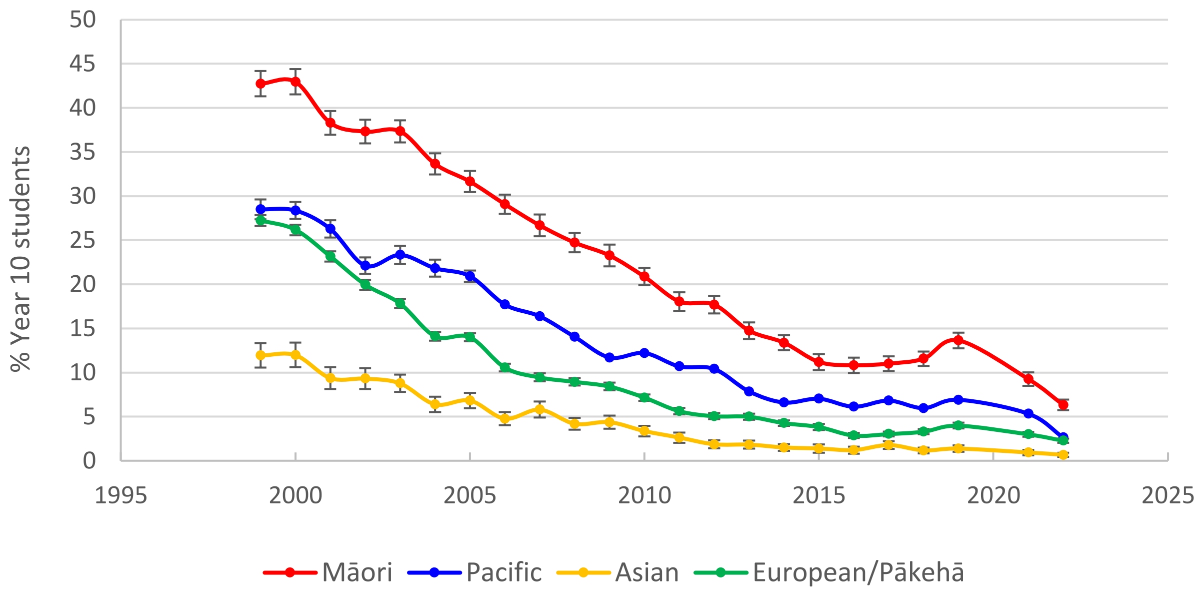

Figure 9: Regular vaping prevalence by ethnicity (2015-2022)

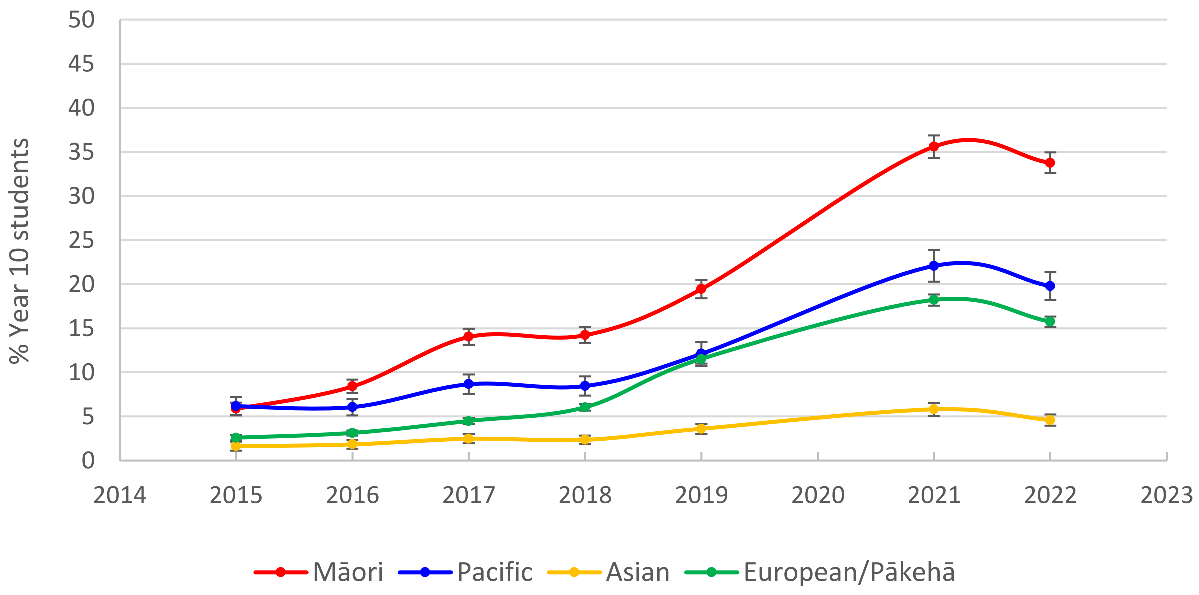

Never smoking increased by a statistically significantly amount for Māori (67.0% in 2021 to 73.8% in 2022), **Pacific** (80.5% to 85.9%), **and European/Pākehā participants** (85.6% to 88.1). When analysed by ethnicity & gender, this was also the case for all groups except for Pacific boys. These were particularly big increases for Māori girls (62.9% to 71.0%) and Pacific girls (78.7% to 86.0%). See Figure 10 and 11.

Ever tried vaping decreased by a statistically significantly amount for Māori (64.0% in 2021 to 61.4% in 2022), **Asian** (19.0% to 16.4%), **and European/Pākehā participants** (40.3% to 37.6%). See Figure 12.

Figure 10: Never smoking prevalence by ethnicity (1999-2022)

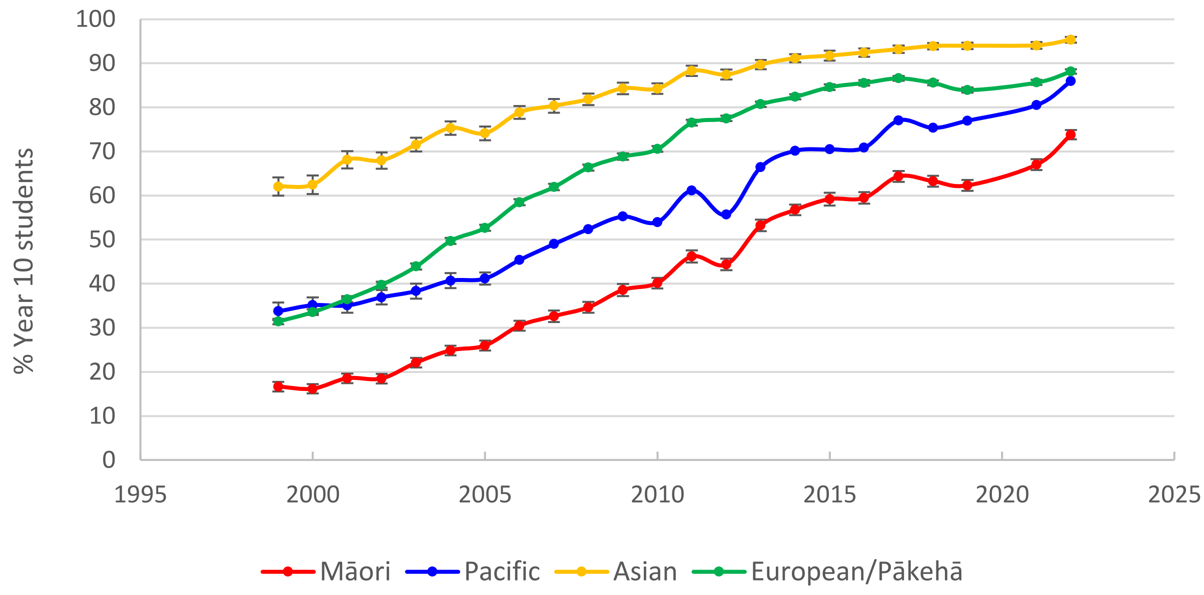

Figure 11: Never smoking prevalence by ethnicity & gender (1999-2022)

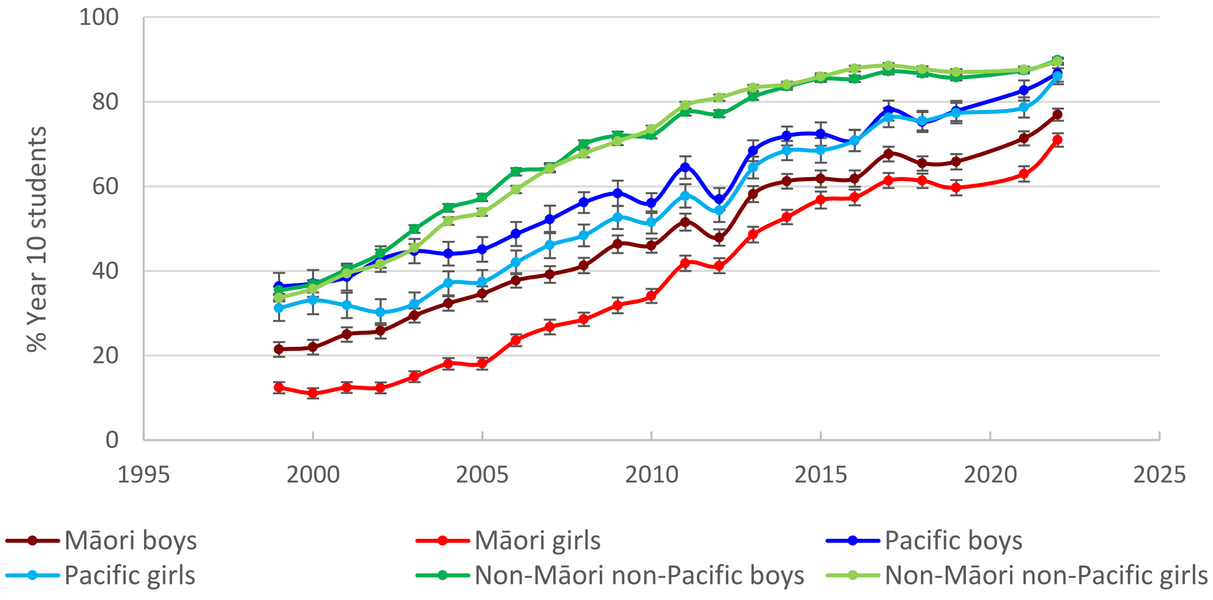

Figure 12: Ever tried vaping prevalence by ethnicity (2014-2022)

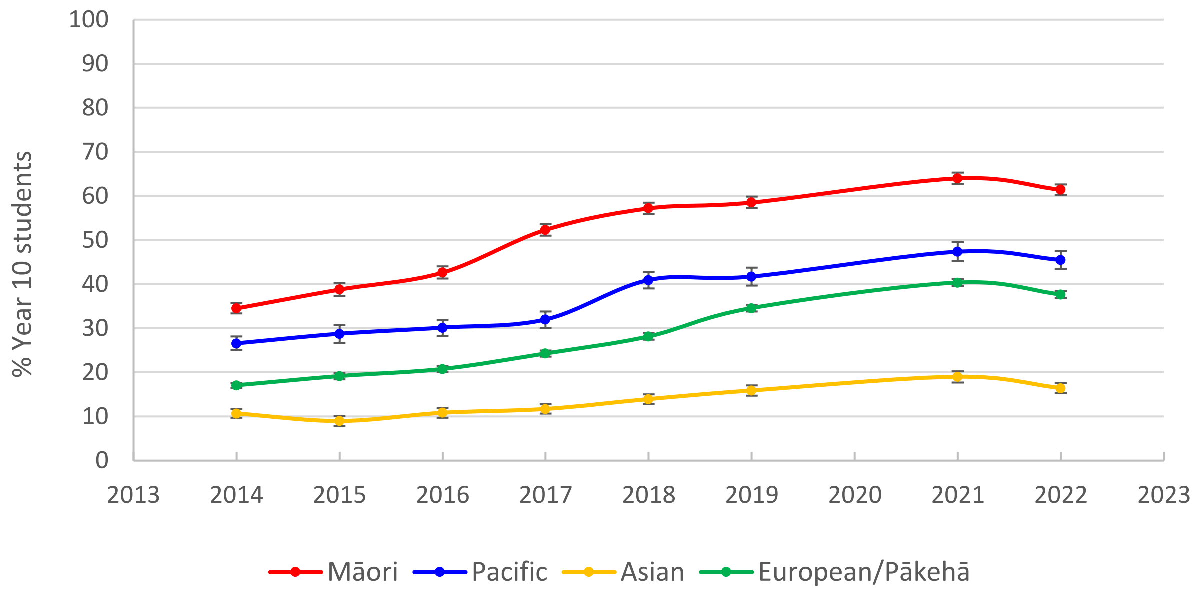

Daily vaping among those who have never smoked increases slightly

Daily vaping among those who have never smoked showed a small but statistically significant increase from 3.1% in 2021 to 4.3% in 2022. There was no statistically significant change in daily vaping rates for those that also smoked daily (86.6% in 2022). See Figure 13.

There was also no statistically significant change in regular vaping rate for never smokers (10.3% in 2022) or daily smokers (95.0% in 2022). See Figure 14.

Figure 13: Daily vaping prevalence by smoking status (2015-2022)

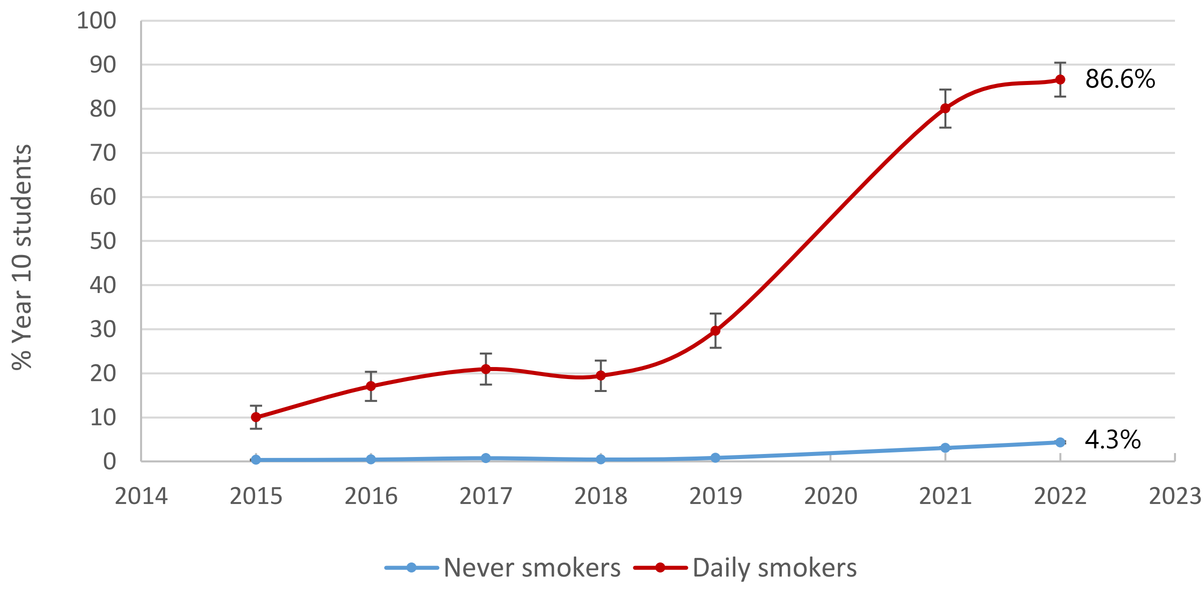

Figure 14: Regular vaping prevalence by smoking status (2015-2022)

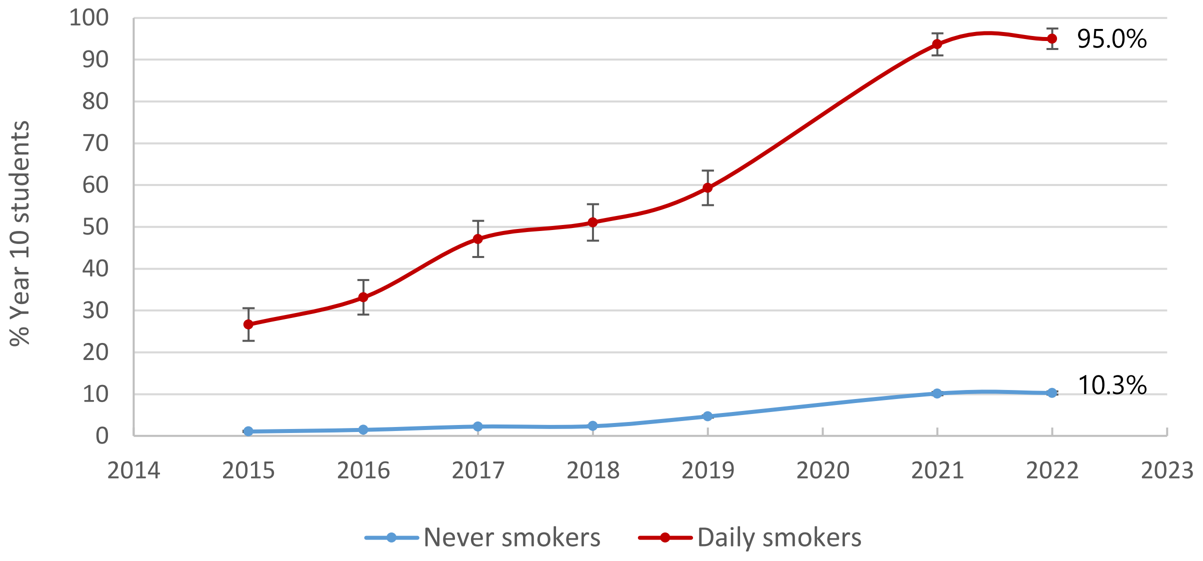

ASH Year 10 Snapshot Survey 2022

	Daily smoking	Daily vaping	Regular smoking	Regular vaping	Never smoking	Ever tried vaping
Total	1.1%	10.1%	3.0%	18.2%	85.8%	40.1%
By ethnicity						
- Māori	2.8%	21.7%	6.3%	33.8%	73.8%	61.4%
- Pacific	1.2%	11.1%	2.6%	19.8%	85.9%	45.5%
- Asian	0.3%	2.3%	0.7%	4.6%	95.3%	16.4%
- European/Pākehā	0.6%	7.6%	2.3%	15.7%	88.1%	37.6%
By ethnicity & gender						
- Māori boys	2.5%	18.3%	5.6%	28.5%	76.9%	56.4%
- Māori girls	3.0%	25.2%	6.8%	39.2%	71.0%	66.5%
- Pacific boys	1.2%	9.3%	2.3%	17.0%	86.7%	41.7%
- Pacific girls	1.2%	12.4%	2.8%	21.8%	86.0%	48.0%
- Non-Māori non-Pacific boys	0.6%	5.5%	1.9%	11.1%	89.9%	31.7%
- Non-Māori non-Pacific girls	0.5%	7.4%	1.9%	15.3%	89.5%	34.3%

Suggested citation

Action for Smokefree 2025 (ASH). 2022. ASH Year 10 Snapshot Survey 2022 Topline – Youth smoking and vaping. Available from: http://ash.org.nz

References

[1]Ministry of Health. Annual Data Explorer 2021/22: New Zealand Health Survey [Data File]. Ministry of Health. Published 2022. Available from https://www.health.govt.nz/
